# Probabilistic human health risk assessment of trace metal exposure in Australian homes: insights from a legacy industrial region

**DOI:** 10.1007/s10653-026-03078-y

**Published:** 2026-03-03

**Authors:** Carlos Ibañez-Del Rivero, C. Marjorie Aelion, Mark Patrick Taylor

**Affiliations:** 1 Faculty of Science and Engineering, School of Natural Sciences, Macquarie University, Sydney, NSW 2109 Australia; 2 Department of Environmental Health Sciences, School of Public Health & Health Sciences, University of Massachusetts Amherst, Amherst, MA 01003 USA; 3 Department of Climate Change, Energy, the Environment and Water, Science and Insights Division, 4 Parramatta Square, 12 Darcy Street, Parramatta, NSW 2150 Australia

**Keywords:** Home contamination, Health risk, Toxic metals, Industrial pollution, Human exposure

## Abstract

Urbanization and industrial activity contribute to environmental contamination, increasing potential health risks for residents via trace metal exposure. This study used a probabilistic human health risk assessment (PHHR) to evaluate trace metal exposure in Australian homes, using garden soil data from 9548 homes and indoor dust samples from 2341 homes. A focused assessment of 75 homes in the historically industrial Illawarra region was included to investigate possible legacy contamination. PHHR captures variability in exposure frequency, contaminant concentrations, and intake rates, unlike deterministic models. Exposure was assessed through ingestion, inhalation, and dermal contact pathways. In Illawarra, garden soils had significantly higher mean concentrations of Cr, Cu, Mn, Ni, and Zn compared to national levels (*p* < 0.05). Indoor dust from older homes and areas near former industrial sites also showed elevated levels of As, Cr, Cu, Mn, Pb, and Zn (*p* < 0.05). Children consistently showed greater health risks than adults, particularly from indoor dust ingestion. Nationally, non-carcinogenic risk (hazard index, HI) exceeded the threshold (HI > 1) above the 98th percentile in soil (HI max = 4.98 children; 3.05 adults) and above the 86th percentile in dust (HI max = 8.48 children; 7.02 adults). Carcinogenic risks (*TCRs*) exceeded 1 × 10⁻^4^ above the 95th percentile in dust. In Illawarra, exceedances were rare (< 0.5%), with HI > 1 observed only above the 99th percentile in dust. These findings highlight indoor dust is a critical trace metal source and the value of PHHR in identifying high-risk cases that may otherwise be overlooked.

**Supplementary Information:**

The online version contains supplementary material available at 10.1007/s10653-026-03078-y.

## Introduction

Gentrification-driven urban development, the repurposing of former industrial areas, and housing expansion near historically contaminated sites have increased the risk of human exposure to pollutants (Holcombe & Keenan, 2020; Keenan & Holcombe, 2021; Radziszewska-Zielina et al., 2022). Legacy and contemporary pollutants can infiltrate home gardens and indoor spaces, posing potential health risks to residents (Entwistle et al., 2019; Gulson et al., 2014; Isley et al., 2022).

The common industrial pollutant lead (Pb) is a well known neurotoxin (Abadin et al., 2020).Exposure to  elevated concentrations of other toxic metal(oid)s (hereafter referred to as trace metals), such as arsenic (As), chromium (Cr(VI)) and nickel (Ni), have been linked to other adverse health effects including lung, respiratory and skin issues (ATSDR, 2016; Fay, 2005; Wilbur et al., 2012). Even essential trace metals such as copper (Cu) and Zn, in high levels, contribute to inflammation and cardiovascular issues (Uriu-Adams & Keen, 2005). Consequently, exposure to elevated levels of these trace metals can pose a significant potential health risk to residents (Ibañez-Del Rivero et al., 2023; Isley et al., 2022).

Previous studies have assessed health risks from indoor dust and garden soils in Australian homes via traditional health risk deterministic models (Gulson et al., 2014; Isley et al., 2022; Taylor et al., 2021). While these approaches provide useful screening-level insights, deterministic models rely on single-point exposure assumptions and do not adequately capture the substantial spatial and behavioral variability inherent to residential environments. In home settings, exposure pathways are influenced by heterogeneous soil and dust contamination, variable building characteristics, and wide inter-individual differences in activity patterns, particularly between children and adults. These factors need more sophisticated assessment approaches. Accordingly, this study applies a Monte Carlo probabilistic human health risk (PHHR) model. This approach addresses parameter variability (e.g., body weight, exposure frequency, and exposure duration) and quantifies uncertainty through repeated simulations and probabilistic risk distributions (Guo et al., 2021).

In addition, comprehensive national-scale health risk assessments for trace metals remain limited. Although the Monte Carlo PHHR modelling approach has been used to assess carcinogenic and non-carcinogenic risks in groundwater and soil, its application has largely been restricted to regional case studies (Dong et al., 2024; Eid et al., 2024; Gaurav & Sharma, 2020; Jiang et al., 2021; Panqing et al., 2023). This study addresses this gap by evaluating trace metal contamination simultaneously in garden soil and indoor dust at a national scale. In addition to providing data from homes across Australia, this study also incorporates a focus on the Illawarra region (New South Wales, Australia) as a historically industrial case study to enable direct comparison with national patterns. The region hosted two major smelting operations: the Port Kembla copper smelter that operated from 1908 to 2003 and the earlier Dapto smelter, located on the western shore of Lake Illawarra and active from 1897 to 1905 (Figure 2) (Yousif, 2012). More broadly, Illawarra’s industrial legacy includes  coal mining, smelting and coke production (Chiaradia et al., 1997; Illawarra Mercury, 2008; Jafari et al., 2020; Markey, 2003; Organ, 1991; Payne et al., 1997; Yousif, 2012). Research on trace metal exposure in Illawarra’s home setting remains limited, with most data confined to government reports and localized Pb studies (Chiaradia et al., 1997; EPA NSW, 2022).

This study investigates contamination in home environments with two key objectives:

- Identify the factors contributing to elevated trace metal concentrations in homes across Australia, with a focus on the Illawarra region.
- Apply a probabilistic human health risk assessment to quantify human trace metal exposure risks in garden soil and indoor dust, facilitating a comprehensive evaluation of potential health impacts.

## Methods and approach

The following section details the methods used to analyze indoor dust and garden soil samples across the national Australian dataset and the Illawarra case study.

### In-home* VegeSafe* and *DustSafe* samples

**Fig. 1:**
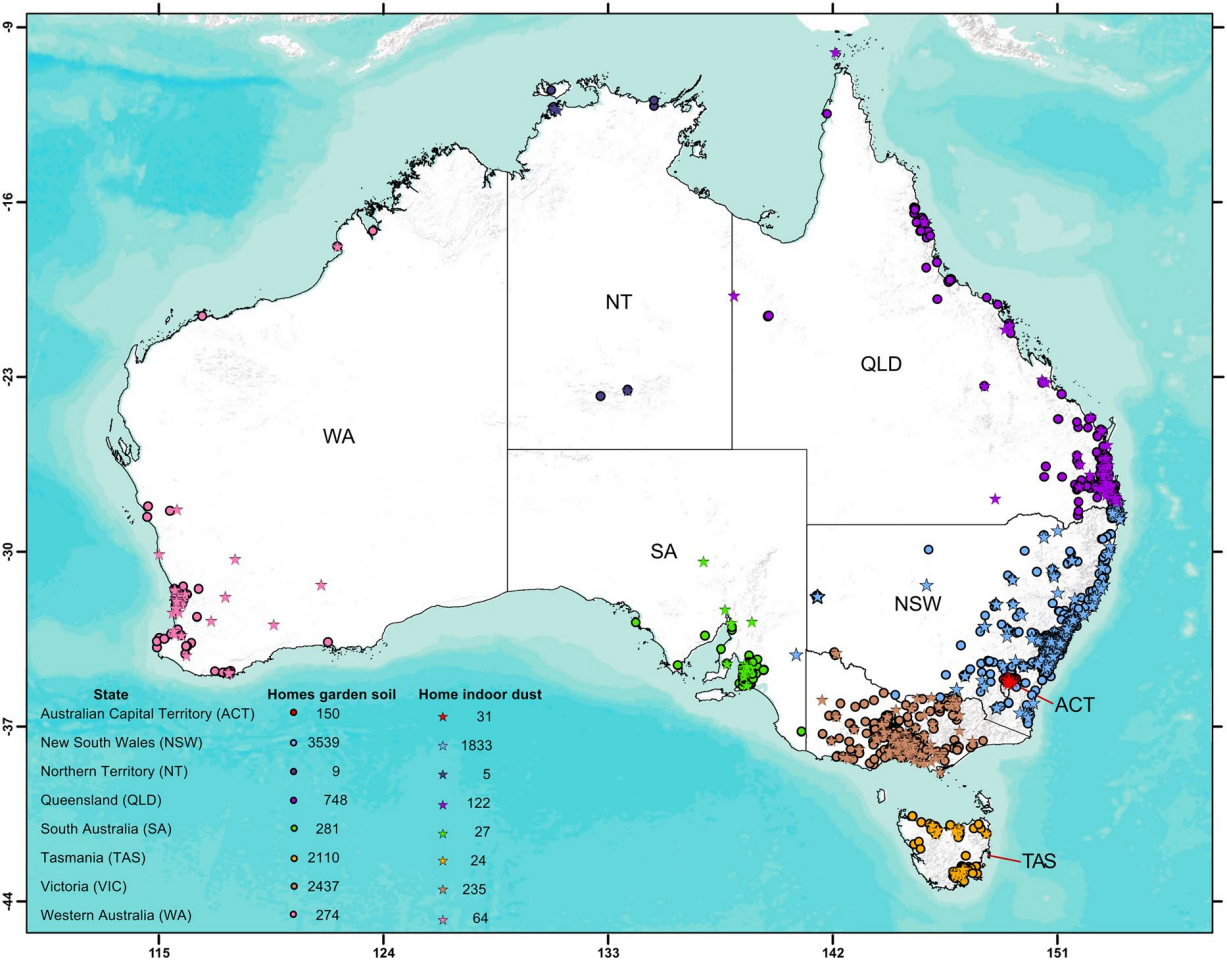
Locations of garden soil samples (9,548 homes; n = 37,488; *VegeSafe)*, and home indoor dust samples (n = 2,341 homes; *DustSafe*) collected from Australian homes. Sample numbers reflect the number of homes; *VegeSafe* typically receives 5 soil samples per home, whereas *DustSafe* requests a single indoor vacuum bag dust sample per household. The concentrations of trace metals measured in these samples were used to undertake probabilistic human health risk assessments

Home garden soil and indoor vacuum cleaner dust samples (hereafter referred to as indoor dust) were collected from Australian homes through the *VegeSafe* and *DustSafe* citizen science programs and posted to Macquarie University in Sydney, Australia (VegeSafe, 2022) for analysis. Participants enrolled voluntarily via the 360 Dust Analysis website (www.360dustanalysis.com). The programs have generated extensive national datasets: *VegeSafe* has operated since 2013 (9,548 homes, n = 37,488 garden soil samples) and *DustSafe* since 2019 (n = 2,341 homes, indoor dust samples). These data were incorporated into the PHHR as detailed in the section “Probabilistic human health risk assessment model in home environments” (Fig. 1).

To ensure consistency, participants received sample instructions for the collection of garden soils (*VegeSafe*) and indoor dust (*DustSafe*) upon enrollment (see also Taylor et al. (2021) and (Isley et al., 2022)). Garden soils were collected using a clean trowel to sample the top 2 cm of soil, which was placed into a labeled resealable bag. Indoor dust was collected by dispensing the contents of a participant’s vacuum cleaner into a labeled resealable bag. Participants received detailed reports summarizing trace metal concentrations in their homes, and recommendations to reduce exposure (Supplementary Figs. S2 and S3).

**Fig. 2:**
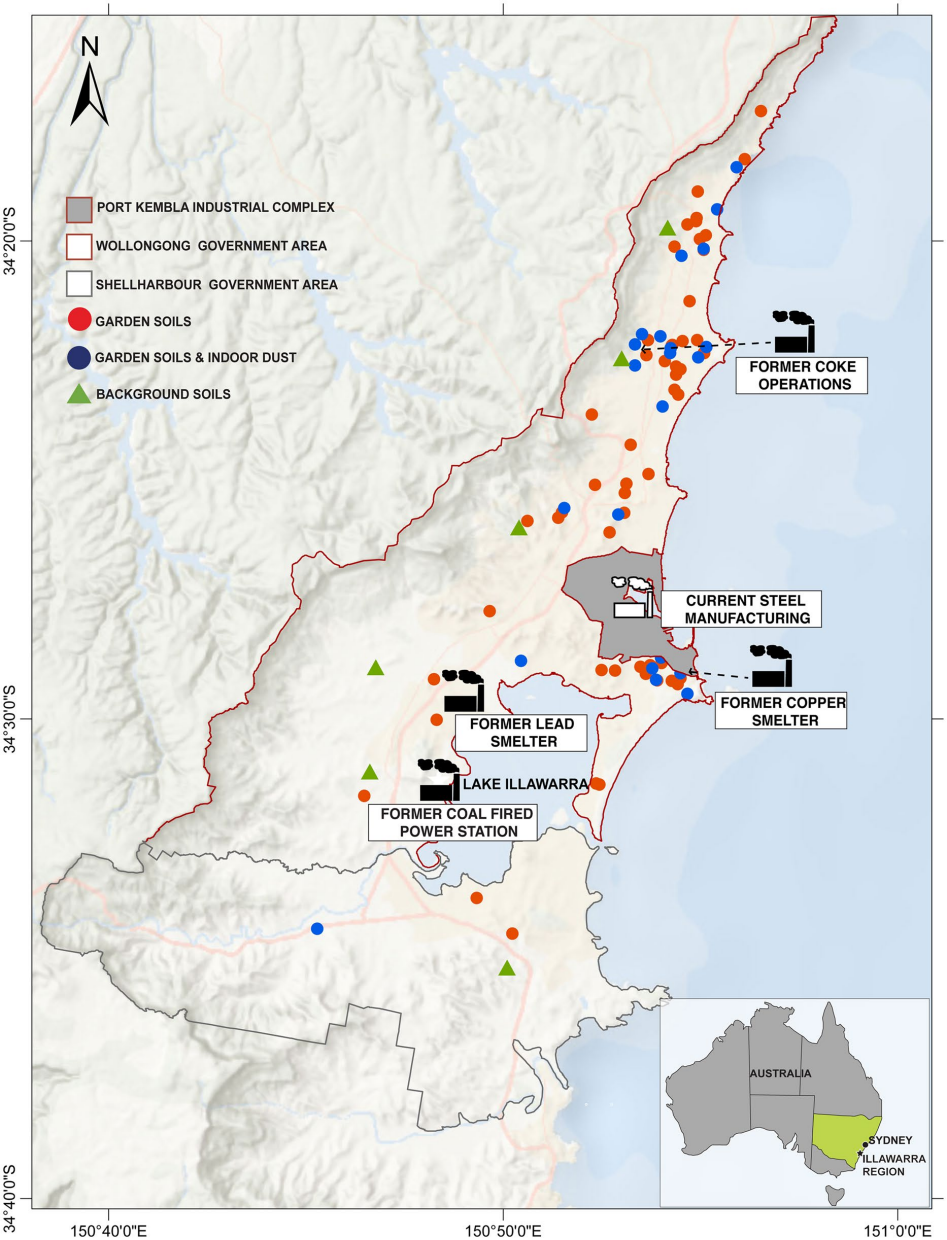
Locations of homes within the Illawarra region, approximately 100 km south of the city of Sydney, New South Wales, Australia. The red circles represent locations with garden soil data (n = 75), and the blue circles represent locations with matched indoor dust and garden soil (n = 23). The green triangles represent locations where background soils were collected (n = 6). The relevant industrial operations are shown. The boundaries of the Illawarra region are divided into Wollongong (red border) and Shellharbour (blue border) boundaries (Australian Bureau of Statistics, 2022)

Concerns about historical contamination and trace metal concentration levels in their home environments led to investigations of the Illawarra region using citizen science engagement between 2021 and 2022. This approach was crucial, as COVID-19 restrictions at the time, limited fieldwork, travel, and direct public interaction (Fig. 2). A total of 356 garden soil samples from 75 home locations (comprising front gardens, back gardens, drip lines, and vegetable gardens) and 23 indoor dust samples (which were home-matched with garden soils) were collected in the Illawarra region. Participants completed a brief questionnaire covering details about their home age and home construction material details, which were investigated to interpret trace metal sources in the Illawarra region (Supplementary Fig. S1). Additionally, trace metal anthropogenic enrichment in home samples was determined by benchmarking against local undisturbed background soil samples (Fig. 2) collected following procedures established in Ibañez-Del Rivero et al. (2023) (Supplementary Table S4). Soil background concentrations were consistent with those reported in nearby areas, except for Mn, which was not referenced (Jafari et al., 2020; Martley et al., 2004).

### Sample trace metal preparation, analysis, and quality assurance

Illawarra garden soils (n = 356), background soils (n = 6), and indoor dust samples (n = 23) were sieved to < 250 µm using stainless steel sieves and an Octagon 200CL sieve shaker. Sieves were cleaned with Milli-Q water and ethanol, then dried at 50 °C for 5 h between samples to prevent cross-contamination. *VegeSafe* home garden soil samples were analyzed as received in bulk form, whereas *DustSafe* indoor dust samples were processed using the same procedure as for the Illawarra samples.

Prepared samples were placed into 35 mm polyethylene X-ray cups sealed with 3.6 μm polyester (Chemplex Mylar) film.

Soil trace metal concentrations were analyzed via a portable X-ray fluorescence (pXRF) Vanta VMW 50 kV fitted with a 4 W tungsten X-ray tube and a silicon drift detector (SDD). The Vanta VMW was operated in soil mode for 20 s per beam (15, 40, and 50 kV), for 60 s, as per *VegeSafe* published methods (Ibañez-Del Rivero et al., 2023; Taylor et al., 2021). Indoor dust samples were analyzed using a pXRF Vanta VMR 50 kV fitted with a 4 W rhodium X-ray tube and an SDD. Measurements were conducted in indoor dust mode for 60 s per beam (15, 40, and 50 kV) for a total of 180 s per *DustSafe* published methods (Ibañez-Del Rivero et al., 2023; Isley et al., 2022). Limits of detection (LODs; mg/kg) for Vanta VMW were: As: 1, Cr: 2, Cu: 2, Mn: 3, Ni: 4, Pb: 1, and Zn: 1. LODs for Vanta VMR (mg/kg) were: As: 1, Cr: 1, Cu: 1, Mn: 3, Ni: 3, Pb: 1, Zn: 1. Concentrations < LODs were assigned a value equal to one half of the corresponding detection limit prior to statistical analysis and Monte Carlo modeling.

Instrumental precision and accuracy were determined using National Institute of Standards and Technology (NIST) standard reference materials (SRMs) equivalent to the sample types analyzed in this study. Soil recoveries for As, Cr, Cu, Mn, Ni, Pb and Zn were determined using the SRM NIST 2709 and NIST 2711a and ranged from 88–113% (x̅ = 96%) and 85–117% (x̅ = 98%), respectively (Supplementary Table S5). Indoor dust recoveries were assessed using NIST SRMs 2583 and 2584 and ranged from 88–124% (x̅ = 109%) and 85–127% (x̅ = 103%), respectively (Supplementary Table S5).

The highest recoveries were observed for As in NIST 2583 and Zn in NIST 2584, both of which have lower certified concentrations than the indoor dust samples analyzed in this study and in comparable investigations (Ibañez-Del Rivero et al., 2024; Isley et al., 2022). Poor recoveries at low analyte concentrations is a recognized characteristic of pXRF analysis and is commonly attributed to reduced signal-to-noise ratios and matrix effects. Despite these elevated recoveries, precision and accuracy remained within acceptable limits based on relative standard deviation and percentage difference criteria, and all data were therefore considered suitable for subsequent analyses (US EPA, 2007; Supplementary Table S5).

### Anthropogenic enrichment in Illawarra homes

Home samples were normalized using enrichment factors (*EFs*) to assess anthropogenic trace metal inputs, following established methods (Barbieri, 2016). The *EF* was calculated as:

$$EF \, = \, TE/RE\_Mn\left( {sample} \right){/}TE\_RE\_Mn\left( {background} \right)$$

where *TE* is the trace metal in the medium studied (e.g., soil), *RE* is the reference trace metal concentration in this case, and *Mn* is deemed the most suitable of the trace metals studied here and as established by others (Ibañez-Del Rivero et al., 2023; Isley et al., 2022). *EF* normalization sets the trace metal background as a reference baseline. Values above this baseline define different degrees of anthropogenic enrichment (Ghrefat et al., 2011; Looi et al., 2019). *EF* ≤ 2 denotes minimal enrichment; *EF* > 2–5 denotes moderate enrichment; *EF* > 5–20 denotes significant enrichment; *EF* > 20–40 denotes very high enrichment; and *EF* > 40 denotes extremely high enrichment (Barbieri, 2016).

### Statistical analysis and trace metal spatial distribution

Garden soil samples from different home locations (*VegeSafe* typically receives 5 samples per home) were averaged to generate a representative home concentration.

Differences between indoor dust and garden soil concentrations were evaluated using Mann Whitney tests for matched Illawarra homes. Associations between trace metal concentrations, home age, and construction materials were assessed using Spearman rank correlations and Kruskal Wallis tests with Dunn’s post hoc comparisons. Non-parametric methods were applied due to non-normal data distributions. Statistical analyses were conducted using Minitab 17.1.0.

Spatial distribution and visualization of trace metal concentrations were performed using ESRI ArcMap 10.8. Graduated symbol maps were used to illustrate spatial patterns, and boxplots comparing indoor and outdoor media were generated using Origin Pro 2019b.

### Probabilistic human health risk assessment model in home environments

Noncarcinogenic health risk (NCHR) evaluates the potential for adverse health effects in a population resulting from chronic exposure to hazardous substances (ATSDR, 2020), whereas carcinogenic risk (CR) estimates the lifetime excess cancer risk associated with such exposure (Taloor et al., 2023). Both NCHR and CR were quantified using Monte Carlo probabilistic modeling implemented in Oracle Crystal Ball.

Monte Carlo model differs from traditional health risk assessment because it incorporates parameter variability and comprehensively explores parameter space rather than fixed values. Furthermore, it provides consistent results through sufficient iterations, providing a more robust understanding of the risk assessments where data may have inherent uncertainty or variability. In environmental analysis, Monte Carlo probability distributions with 10,000 iterations have been suggested to enable a thorough exploration of the entire parameter space, capturing the full range of potential outcomes (Panqing et al., 2023; Yang et al., 2019).

Risks were evaluated for two age groups, children (2 years old) and adults, under an Australian exposure scenario (Supplementary Table S8). Modeled inputs included trace metal concentrations in garden soils and indoor dust, supplemented with *VegeSafe* and *DustSafe* datasets. Inhalation, dermal contact, and ingestion exposure pathways were considered, with detailed equations and parameters provided in the supplementary materials (Supplementary S6, S7).

### Parameters sensitivity analysis

A probabilistic sensitivity analysis was conducted using the *DustSafe* dataset to identify key contributors to variability in the NCHR hazard index (HI) for children exposed to indoor dust. Monte Carlo simulations (n = 10,000 iterations) were performed in R Studio (Version 2025.09.0) , incorporating variability in trace metal concentrations and exposure parameters. HI values were calculated for each iteration using route-specific reference doses (RfDs). Spearman rank correlations between input parameters and HI values were visualized using a tornado plot to illustrate the relative contribution of each parameter to HI variability.

## Results and discussion

The results and discussion detailed below evaluate the contamination risks posed to humans from garden soils and indoor dust across Australian homes and the Illawarra region.

### Trace metal levels in home environments

Home garden soil trace metal concentrations in the investigated homes of the Illawarra region were benchmarked against the (a) Australian National Environment Protection Agency (NEPM) Health Investigation Level A guideline (HIL–A) for accessible residential soil (NEPC, 2013) and (b) the *VegeSafe* Australian national garden soil database mean concentrations (Table 1). For the suite of trace metals, As, Cu, Mn and Ni, Illawarra garden soil mean concentrations in all homes were below their respective HIL-A values. However, Cr, Pb and Zn HIL-A values were greater in  1, 4 and 1 homes, respectively (Table 1). Furthermore, the Illawarra garden soil mean concentrations of Cr (51 mg/kg), Cu (137 mg/kg), Mn (477 mg/kg), Ni (13 mg/kg) and Zn (442 mg/kg) were greater than the *VegeSafe* Australian national mean concentrations (47 mg/kg, 49 mg/kg, 254 mg/kg, 11 mg/kg, and 314 mg/kg, respectively) (*p* < 0.05). The Illawarra garden soil As (11 mg/kg) and Pb (143 mg/kg) mean concentrations were equal to or lower than the *VegeSafe* Australian national mean soil concentrations (11 mg/kg and 196 mg/kg, respectively) (*p* < 0.05) (Table 1).

**Table 1:** Descriptive statistics of trace metal concentrations (mg/kg) in Illawarra region garden soils (n = 356), indoor dust (n = 23), and background soils (n = 6) compared with the *VegeSafe* and *DustSafe* databases

	As	Cr	Cu	Mn	Ni	Pb	Zn
**Garden soil n = 356 (75 homes)**							
Mean	11	51	137	477	13	143	442
95CI U	13	57	205	546	19	196	714
95CI L	8	45	70	407	7	91	171
5	1	22	25	195	1	14	64
25	4	35	39	301	4	33	145
50	8	51	53	395	8	82	242
75	15	60	88	549	13	171	401
95	32	92	588	1105	28	361	829
Min	1	14	17	159	1	3	4
Max	52	164	1752	1866	206	1644	9697
SD	10	24	278	289	25	216	1115
**(a) NEPM HIL-A**	100	100	7000	3000	400	300	8000
**Indoor dust n = 23**							
Mean	33	189	283	503	55	245	1562
95CI U	43	252	376	592	66	357	1887
95CI L	23	120	198	400	43	123	1312
5	6	83	110	279	20	58	652
25	17	95	185	377	36	76	1038
50	27	138	240	423	52	162	1410
75	41	174	301	578	69	273	1984
95	84	596	469	928	86	533	2695
Min	4	69	84	212	16	47	587
Max	88	667	1166	1177	139	1364	2912
SD	24	159	214	230	27	282	670
**(b) *VegeSafe* Australian garden soils (n = 37,488)**							
Mean	11	47	49	254	11	196	314
> HIL-A*	0	1*	0	0	0	4*	1*
**(c) *DustSafe* Australian indoor dust (n = 2,341)**							
Mean	43	119	240	297	64	402	1619
> HIL-A*	0	16*	0	0	0	6*	

> HIL-A*: represents the number of homes exceeding their corresponding NEPM HIL-A guideline in the media

95% CI U—Upper 95% confidence interval, based on the mean value

95% CI L—Lower 95% confidence interval, based on the mean value

In the absence of guidelines, home indoor dust trace metal concentrations were benchmarked to the residential soil NEPM HIL-A guideline since a portion of the indoor dust mass (~ 60%) is estimated to be sourced from outdoors (Ibañez-Del Rivero et al., 2023). Additionally, the mean indoor dust trace metal concentrations were benchmarked against the *DustSafe* Australian national indoor dust database mean concentrations (n = 2,341) (Table 1). Among the trace metals studied, Cr and Pb exceeded their corresponding NEPM HIL-A guidelines at 16 and 6 locations, respectively (Table 1). However, Cr concentrations in this study were determined in total, which likely overestimate Cr exceedances as guidelines are based on Cr(VI). Similarly, the mean indoor dust concentrations of Cr (189 mg/kg), Cu (283 mg/kg), and Mn (503 mg/kg) in Illawarra homes were higher (p < 0.05) than the *DustSafe* Australian national mean concentrations (119 mg/kg, 240 mg/kg, and 297 mg/kg, respectively). By contrast, As (33 mg/kg), Ni (55 mg/kg), Pb (245 mg/kg), and Zn (1562 mg/kg) concentrations were lower than the *DustSafe* Australian national mean concentrations (43 mg/kg, 64 mg/kg, 402 mg/kg, and 1619 mg/kg, respectively) (*p* < 0.05) (Table 1).

The relationship among home age, construction materials, and elevated trace metal concentrations in garden soils and indoor dust were investigated in the Illawarra region, and comparisons with findings from major Australian cities were drawn. Unlike major Australian cities, analysis of the data revealed that construction materials were not significantly correlated with residential trace metal concentrations in any medium (Supplementary Table S9) in Illawarra homes (Doyi et al., 2019; Ibañez-Del Rivero et al., 2023; Isley et al., 2022; Taylor et al., 2021).

While home age was not associated with trace metal concentrations in garden soils (*p* < 0.05), it was moderately correlated with As, Pb, and Zn levels in indoor dust samples (rs = 0.573, 0.471, 0.533, respectively; *p* < 0.05) (Supplementary Table S10). This suggests that older homes may contribute to higher concentrations of As, Pb, and Zn in indoor dust, likely implicating indoor sources. For example, Pb and Zn can originate from paint pigments in older homes, Ni from degraded alloys, and As and Cr from wood preservatives and are generally present in the materials of older homes (Isley et al., 2022; Rasmussen et al., 2018).

In addition, a comparison between home-matched garden soil and indoor dust samples revealed higher trace metal concentrations indoors for all metals except manganese (Mn), supporting previous studies emphasizing the tendency for indoor environments to have higher concentrations of contamination than outdoor environments (Doyi et al., 2019; Ibañez-Del Rivero et al., 2023; Isley et al., 2022; Rasmussen et al., 2018). Homes constructed before the 1970s in Australia often contained elevated concentrations of Pb due to historic Pb-based paint usage and leaded petrol (Doyi et al., 2019; Gulson et al., 1995; Ibañez-Del Rivero et al., 2023). Furthermore, older Australian homes exhibit more vulnerabilities, such as draft issues and moisture damage, allowing for the infiltration of airflow and particlescompared to newer homes that are built with stricter construction practices adopting energy efficiency regulations introduced in 2001 (CSIRO, 2022). The ability of metals to adhere to soot and finer particle-size fractions has also been identified as a mechanism contributing to increased indoor trace metal concentrations (Berasaluce et al., 2019; Ibañez-Del Rivero et al., 2023; Rasmussen et al., 2008). By contrast, there was no significant difference in Mn concentrations between interior and exterior samples (Supplementary Fig. S11). Unlike the other trace metals measured in this study, Mn sources are associated with pedogenesis, have an affinity for outdoor soils, and lack indoor sources (Ha et al., 2014; Rasmussen et al., 2018).

### Spatial distribution of trace metals in the Illawarra region

The distribution of trace metals in indoor dust and garden soils across the Illawarra region reflects the area's industrial history, with distinct patterns emerging based on proximity to former industrial operations. Elevated concentrations of As, Ni, and Cr in garden soils near former coke operations (Supplementary Fig. S12) suggest a shared source linked to coal and coke combustion, a historically prominent industry in the region (Bartoňová & Raclavská, 2022; Cai et al., 2021; Tavares et al., 2022). Notably, Cr concentrations were particularly pronounced, which is consistent with its role as a byproduct of coal combustion (Shah et al., 2012; Wang et al., 2022). By contrast, Cu, Pb, and Zn in garden soils showed a strong spatial association with the Port Kembla industrial complex. This pattern is consistent with historical Cu smelting, as evidenced by elevated levels reported in public land soils and sediments (Jafari et al., 2020; Payne et al., 1997), and contemporary steel manufacturing, which likely contributed to localized Zn depositions (Supplementary Fig. S13). The latter claim is supported by research indicating the spread of Zn in nearby aquatic bodies (Jones et al., 2019). Copper (Cu) concentrations were notably elevated around Port Kembla in indoor dust and garden soils, whereas the Pb concentrations were high near coke operations and Port Kembla, which is consistent with historical smelting and leaded petrol emissions (Chiaradia et al., 1997). Zinc (Zn) concentrations were uniformly elevated in indoor dust, likely due to indoor sources, while garden soil Zn concentrations were relatively high near Port Kembla, aligning with slag deposits across the Lake Illawarra catchment fromhistorical smelting activities affecting proximate homes (Jones et al., 2019) (Supplementary Fig. S12). These spatial trends suggest that coal combustion, smelting, and steel production are key contributors to trace metal contamination in the region. By contrast, Mn industrial sources have not been identified in the area, and sediment investigations in Lake Illawarra have been linked to elevated concentrations from rocks and soil weathering and erosion sources (Chenhall et al., 1994).

Furthermore, the mean EF normalization revealed distinct enrichment patterns, with garden soils showing a decreasing order of Pb > Zn > Ni > Cu > As > Cr and mean indoor dust following a decreasing order of Ni > Zn > Pb > Cu > As > Cr metal sources (Supplementary Fig. S14, Supplementary Table S15). The contrasting enrichment patterns, with Ni being more elevated indoors and Pb more enriched in garden soils, indicate distinct sources and environmental migration pathways. This increase in Ni levels in indoor dust is consistent with emissions of fly ash from the former power generation plant in the Illawarra region (Chenhall et al., 1994; Chiaradia et al., 1997; Payne et al., 1997). Additional sources of Ni include vehicular and industrial emissions, which are often associated with fine particle fractions (10–100 µm) that can infiltrate indoor environments (Berasaluce et al., 2019; Bourliva et al., 2017).

### Probabilistic human health risks in Australian and Illawarra homes

#### Non-carcinogenic health risk

The results of the modeled probabilistic NCHR for the Illawarra region and Australian homes indicated mean hazard quotient (HQ) values in the decreasing order of ingestion > dermal > inhalation for garden soils and indoor dust, with higher values observed in children than in adults (*p* < 0.01) (Supplementary Table S16). This pattern aligns with established exposure pathways, where ingestion dominates due to common hand-to-mouth behaviors in children and incidental soil ingestion (enHealth, 2012).

Furthermore, the estimated HQ mean values in the modeled age groups were greater for children than for adults for all exposure routes from indoor dust and garden soils (*p* < 0.01) (Supplementary Table S16). The higher HQ values observed in children is attributed to their high ingestion and inhalation rates and low body weight according to the parameters proposed by NEPC (2011) and enHealth (2012) (Supplementary Table S8).

**Fig. 3:**
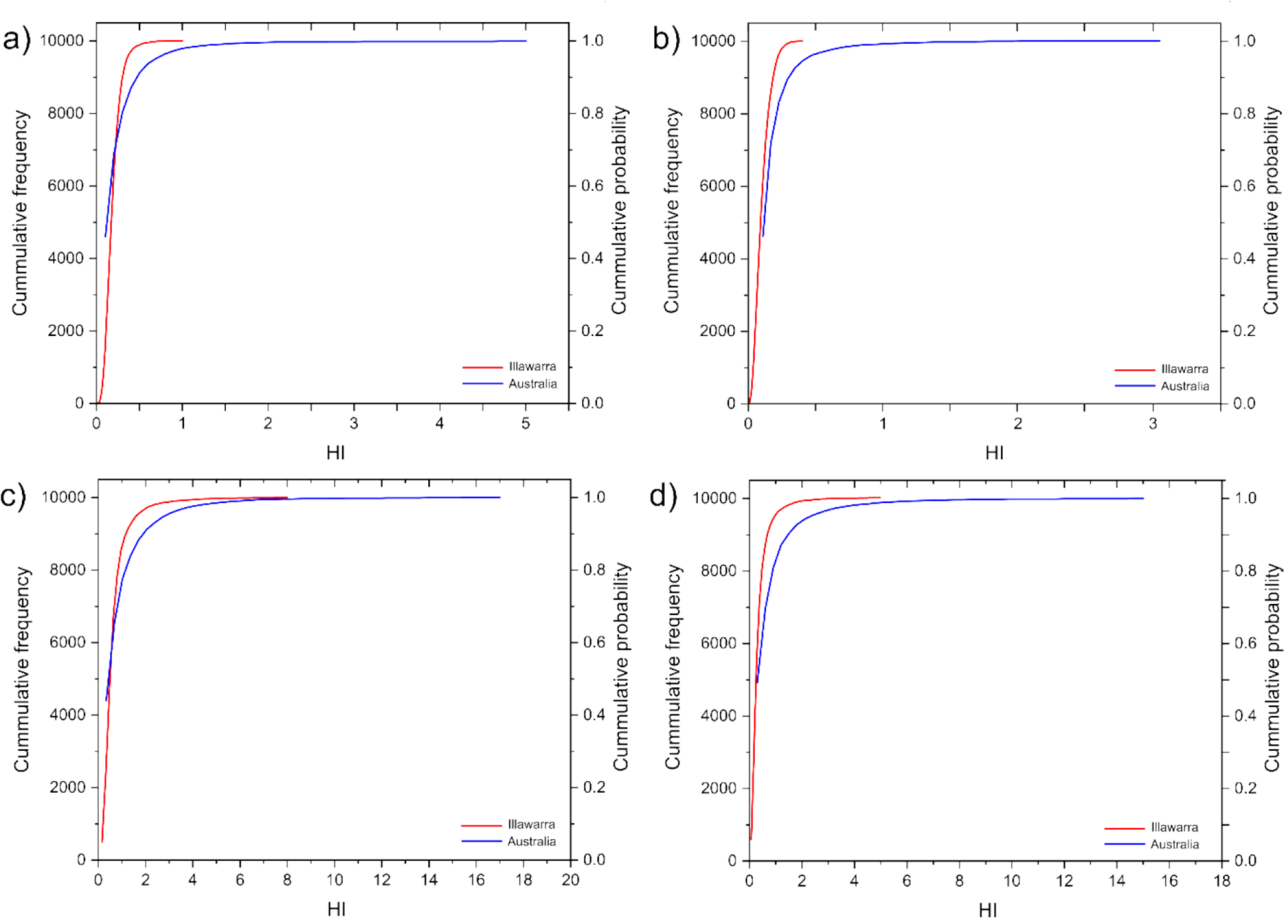
Cumulative frequency and probability distributions of NCHR, which are based on 10,000 iterations, for the Illawarra region and Australian homes. The modeled data include exposure from **a)** garden soil for children, **b)** garden soil for adults, **c)** indoor dust for children, and **d)** indoor dust for adults. The cautionary risk is established at HI = 1

The modeled mean HI for Australian homes exceeded that of the Illawarra region across all exposure routes and age groups (Supplementary Table S16). This pattern was observed throughout the probabilistic distribution of HI values modeled for Australian homes, which presented higher cumulative probabilistic values than the modeled data for the Illawarra region (Fig. 3).

While the mean HI values remained below the threshold of concern (HI = 1), instances of HI > 1 were observed in the upper percentiles of the distribution. For garden soil exposure in Australian homes, HI > 1 occurred above the 98th percentile for children (HI maximum = 4.98) and adults (HI maximum = 3.05). Similarly, for indoor dust exposure, HI > 1 was observed above the 86th percentile for children (HI maximum = 8.48) and the 94th percentile for adults (HI maximum = 7.02) (Fig. 3). These results suggest that although the majority of the population faces minimal risk, a small subset, particularly children, may experience elevated exposure levels in high-risk scenarios.

In the Illawarra region, HI > 1 occurred only above the 99th percentile for indoor dust exposure (maximum = 1.48 in children; 1.30 in adults). These cases were rare (< 0.5% of iterations) and negligible (Fig. 3). The lower HI values in Illawarra than in Australian homes may reflect regional differences in trace metal concentrations, exposure factors, and the influence of localized industrial activities.

Higher HI values for indoor dust indicate that indoor environments are key source of trace metal exposure, especially for children. Addressing contamination in older homes and industrial areas including dust sources from degraded materials and airborne particulates, is essential to reducing exposure risk.

#### Carcinogenic health risk

Lifetime carcinogenic risk calculations were modeled for As and Pb, as these are the only trace metals from the suite of elements studied with established slope factors in toxicological databases from official agencies (IRIS, 1985; OEHHA, 2009). Compared with the NCHR estimates, the mean values of the carcinogenic effects were consistently higher in children than in adults (Supplementary Table S17). This elevated risk in children was observed across all exposure pathways and in the Illawarra and Australian models alike, including indoor dust and garden soils, for As and Pb (Supplementary Table S17). Moreover, the mean cumulative risk values were greater in children than in adults when considering each exposure pathway individually (e.g., ingestion) and when assessing the combined risk of all exposures or total risk (Supplementary Table S17).

The results for lifetime carcinogenic risk indicated that the mean values were consistently below the level of concern (< 1 × 10^−4^) for indoor dust and garden soils across the modeled age groups in the Illawarra and Australian homes (Supplementary Table S17).

**Fig. 4:**
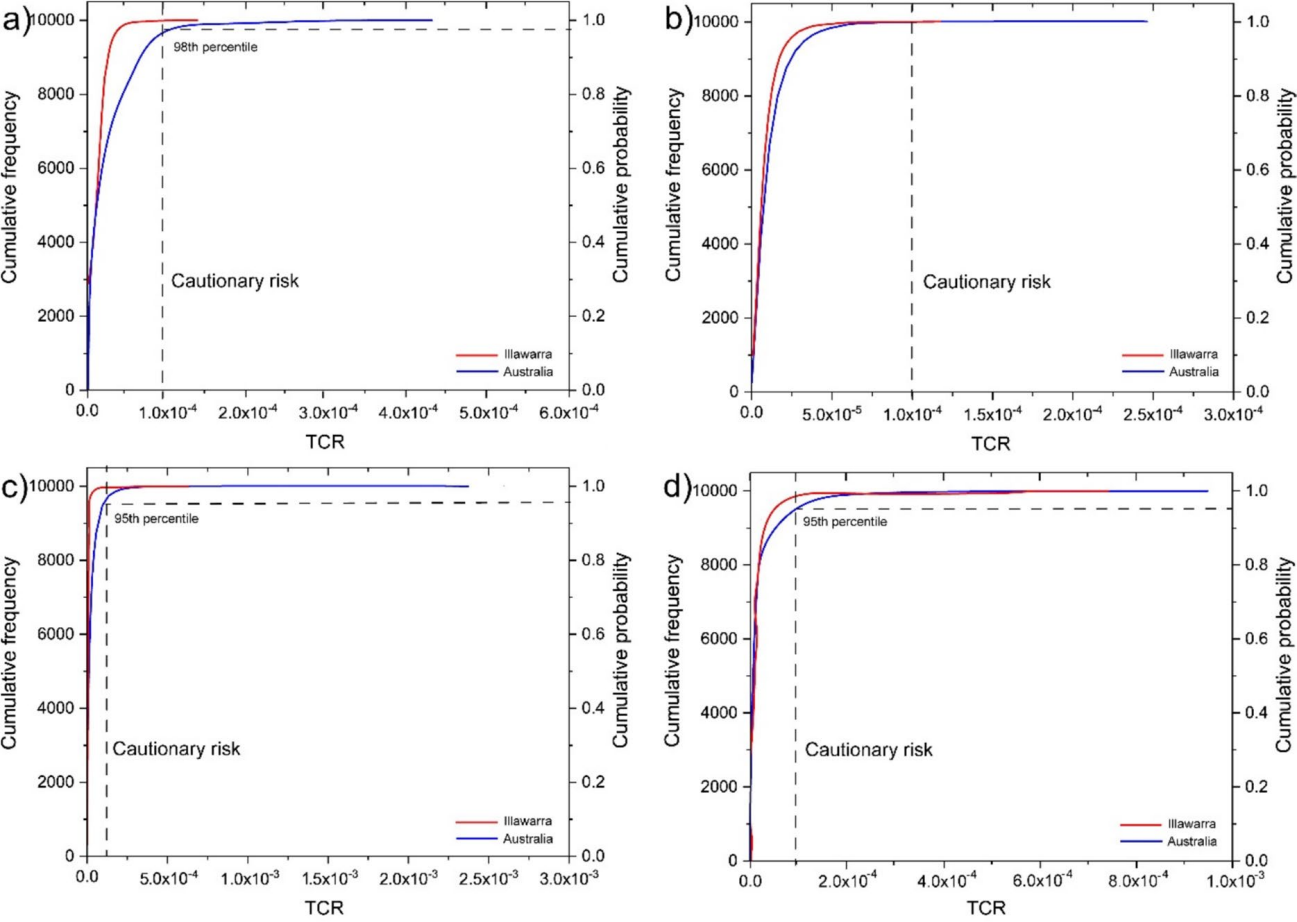
Cumulative frequency and probability distributions of total carcinogenic risk (TCR), which are based on 10,000 iterations, for the Illawarra region and Australian homes. The modeled data include exposure from **a)** garden soil for children, **b)** garden soil for adults, **c)** indoor dust for children, and **d)** indoor dust for adults. Cautionary risk is established at TCR = 1 × 10^−4^

However, the cumulative probability distributions revealed instances where the total carcinogenic risk (TCR) exceeded the level of concern. For garden soil exposure in Australian homes, TCR values > 1 × 10^−4^ were observed above the 98th percentile for children (TCR maximum = 4.42 × 10^−4^). Similarly, for indoor dust exposure, TCR values > 1 × 10^−4^ were noted above the 95th percentile for children (TCR maximum = 2.38 × 10^−3^) and adults (TCR maximum = 9.65 × 10^−4^) (Fig. 4).

Several key factors must be considered when interpreting these results. First, carcinogenic effects are likely to occur after chronic exposure and bioaccumulation over a lifetime; short-term or occasional exposure to low levels of As and Pb is not generally considered to cause a significant health risk. Nevertheless, exposure should still be minimized, particularly for Pb due to its well-established toxicity (Tan et al., 2016; Tchounwou et al., 2012). Second, the risk assessment model used follows a highly conservative approach, assuming 100% bioavailability of trace metals, a likely overestimation in most cases. However, a conservative scenario ensures that even small concentrations of trace metals are considered, and it is a protective measure, providing that any potential risks are not underestimated (Belluck et al., 2003).

#### Parameters sensitivity analysis

**Fig. 5:**
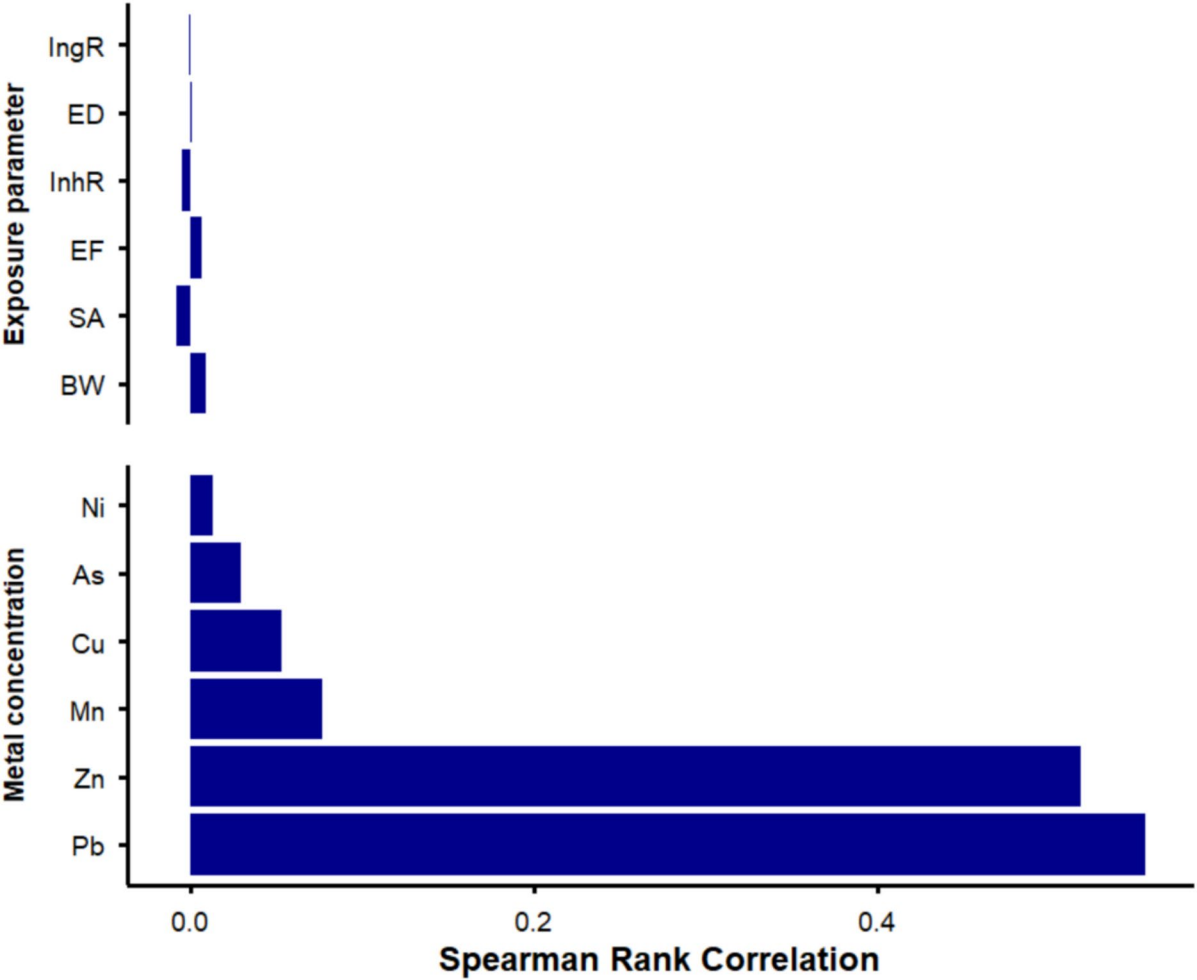
Sensitivity analysis of HI for children’s indoor dust exposure in Australian homes (*DustSafe* dataset). Spearman rank correlations were used to assess the influence of input parameters, including metal concentrations and exposure factors, on modeled HI values. IngR: ingestion rate, ED: exposure duration, InhR: inhalation rate, EF: exposure frequency, SA: exposed dermal area, BW: body weight

The sensitivity analysis, based on Spearman rank correlations, indicated that trace metal concentrations particularly Zn and Pb were the strongest contributors to variability in HI values for indoor dust exposure (Fig. 5). In contrast, exposure parameters such as ingestion rate (IngR), exposure frequency (EF), and body weight (BW) had comparatively smaller influence on overall risk estimates. These results suggest that contaminant levels in indoor dust are the primary drivers of modeled health risk, underscoring the importance of mitigating indoor dust contamination in high-risk environments.

#### Contextualizing probabilistic human health risk results

Compared with deterministic methods that rely on single-point estimates or worst-case scenarios, incorporating variability allows for more realistic risk calculations. However, few studies have applied probabilistic risk assessment to urban environmental samples, which limits direct comparability with this study. In this limited number of investigations, an urban soil study in Udupi, India an industrial city, reported comparable contamination profiles for Pb, Cr, Ni, and As, with significant non-carcinogenic risks (HI > 1) identified for children using Monte Carlo modeling. Carcinogenic risks for As were also within the range of concern (1 × 10^−4^ to 3 × 10^−4^ for adults and 1 × 10^−4^ to 2 × 10^−3^ for children) (Shetty et al., 2024). A study conducted on road dust in Anyang, China, an area reliant on the steel and coal industries, identified elevated concentrations of Cu, Mn, Pb, and Zn. Research has indicated that non-carcinogenic risks for children exceed safe thresholds (HI = 2.2, range: 0.42–22.3), although carcinogenic risks remain within acceptable limits (TCR ~ 10^−5^) (Han et al., 2023). These findings highlight that industrial legacy sites can exhibit elevated trace metal concentrations, with children being particularly vulnerable to non-carcinogenic  exposure risks. By comparison, the Illawarra region did not exhibit widespread pollution concerns, except in localized homes near legacy industrial sources such as Port Kembla. The larger Australian dataset produced higher HI and TCR values, with a greater cumulative probability of exceeding the levels of concern. This is likely because many samples were collected from densely populated inner-city areas of Australian capital cities where pollution is more concentrated because of historical and ongoing urban and industrial activities.

In response to industrial legacy contamination in Port Kembla, the New South Wales Environment Protection Authority (NSW EPA) recently reached an agreement with the former owners of the Port Kembla copper smelter to fund an 18 million AUD cleanup effort focused on remediating Pb contamination in roof dust and soil in hundreds of nearby homes (Chandler, 2025). This indicates that although the Illawarra region exhibits a lower overall risk compared with major Australian cities, localized health risks persist in homes adjacent to former industrial facilities.

 The *VegeSafe* national survey of trace metals in garden soils (n = 17,256), based on a deterministic health risk approach, identified elevated Pb concentrations, with non-carcinogenic risks (HQ > 1) for children in older inner-city areas of Melbourne and Sydney, Australia’s largest cities (Taylor et al., 2021). On the other hand, the *DustSafe* national survey of Australian indoor dust (n = 1,310), which similarly employed a deterministic health risk approach, reported no significant non-carcinogenic (NCHR) or total carcinogenic (TCR) risks below thresholds of concern (HI < 1; TCR < 1 × 10^−4^) (Taylor et al., 2021).

Building on previous deterministic assessments, the probabilistic modeling approach applied in this study enabled evaluation of upper-percentile scenarios, capturing exceedances in the NCHR (expressed as HI) and TCR, particularly for children exposed through indoor dust ingestion in Australian homes. By incorporating exposure variability and parameter uncertainty, this approach identified high-risk cases that may not be apparent under single-point deterministic estimates  and further emphasized indoor dust as a critical exposure pathway in highly polluted homes, underscoring the need for more refined risk assessment strategies.

## Study limitations

Several limitations should be considered when interpreting the findings of this study. The Illawarra dataset included a relatively small number of samples, particularly for home‑matched indoor dust (n = 23). The small sample size for Illawarra reduces statistical power compared with the larger Australian dataset. Therefore, the results should be viewed as indicative rather than definitive, and caution is warranted when generalizing these findings.

Indoor dust concentrations were benchmarked against the NEPM HIL‑A guideline for residential soils due to the absence of dust‑specific regulations. Although this approach is supported by evidence that a substantial proportion of indoor dust originates from outdoor soil (~ 60%), it may overestimate exceedances for certain metals. This is especially relevant for Cr, as existing soil guidelines apply to Cr(VI) whereas this study measured total Cr. Consequently, exceedances should be interpreted conservatively.

The PHHR modelling applied a conservative framework that assumed 100% bioavailability of trace metals and used upper‑bound exposure scenarios for ingestion, inhalation, and dermal contact. While protective and widely used in screening‑level assessments, this approach is likely to overestimate actual risks for residents.

Overall, these limitations indicate that, while the study provides a robust screening‑level assessment of large‑scale trace metal contamination and associated human health risks, further research is needed particularly studies incorporating larger numbers of home‑matched indoor dust samples and targeted investigations in high‑risk areas.

## Conclusions

In relation to the study aims, the following results were observed:

1. Home age was linked to more elevated concentrations of As, Pb and Zn in indoor dust, whereas, garden soils showed no such pattern, implying the presence of additional indoor sources within homes. Furthermore, homes near industrial sites presented elevated trace metal levels. Several homes near industrial areas contained garden soil (n = 4) and indoor dust (n = 6) Pb levels above the NEPM HIL-A guidelines.
2. Probabilistic human health risk models revealed that overall risks were low, but children consistently exhibited higher susceptibility than adults, particularly through ingestion of indoor dust. Although most homes fell below thresholds of concern, a small proportion of Australian homes exceeded upper‑percentile risk levels for children, driven largely by variability in indoor dust Pb and Zn concentrations.

This approach captures upper-percentile exceedances, particularly for children exposed to indoor dust, identifying high-risk cases that may otherwise be overlooked. These findings reinforce the importance of indoor dust as a critical exposure pathway and emphasize the need for refined risk assessments and future studies targeted at strategies to protect vulnerable populations in highly polluted environments.

## Supplementary Information

Below is the link to the electronic supplementary material.

Supplementary file1 (DOCX 8607 kb)

## Data Availability

Data are provided within the manuscript or supplementary information files.
