# Supplementary material for: Probabilistic human health risk assessment of trace metal exposure in Australian homes: insights from a legacy industrial region
Source: Environ Geochem Health. 2026 Mar 3;48(5):210. doi: 10.1007/s10653-026-03078-y (PMC12953340; doi:10.1007/s10653-026-03078-y)
Supplement: Supplementary file 1 — Supplementary file1 (DOCX 8607 kb) [file 10653_2026_3078_MOESM1_ESM.docx]

**Supplementary Material**

**Probabilistic Health Risk Assessment of Trace Metal Exposure in Australian Homes: Insights from a Legacy Industrial Region**

Carlos Ibañez del Rivero^1^, C. Marjorie Aelion^2^, Mark Patrick Taylor^1,3*^

^1^School of Natural Sciences, Faculty of Science and Engineering, Macquarie University, Sydney, New South Wales, 2109, Australia

^2^Department of Environmental Health Sciences, School of Public Health & Health Sciences, University of Massachusetts Amherst, Amherst, Massachusetts 01003, United States

^3^Environment Protection Authority Victoria, EPA Science, Centre for Applied Sciences, Ernest Jones Drive, Macleod, Melbourne, Victoria, 3085, Australia

^*^Corresponding author: Carlos Ibañez del Rivero

Email: [carlos.ibaez-del-rivero@hdr.mq.edu.au](mailto:carlos.ibaez-del-rivero@hdr.mq.edu.au)

This supplementary comprises 25 pages, 13 Tables, 7 Figures.

**Page S3 – S4: Supplementary Figure S1.** Questionnaires participants home characteristics.

**Page S5: Supplementary Figure S2.** Report DustSafe participants receive outlining the concentration of trace metals in their home environment.

**Page S6: Supplementary Figure S3.** Report VegeSafe participants receive outlining the concentration of trace metals in their home environment.

**Page S7: Supplementary Table S4.** Concentrations of the six soil profiles to determine the background of the study area.

**Page S8: Supplementary Table S5.** Detailed quality control of SRMs and sample replicates.

**Page S9 – S10: Supplementary S6.** Equations for human health risk calculations.

**Page S11: Supplementary Table S7.** Reference doses (RfD) and Cancer Slope Factors (CSF) for the risk calculation of the various trace metals studied.

**Page S12: Supplementary Table S8.** Toxicological parameters and exposure factors for risk calculations

**Page S12: Supplementary Table S9.** Kruskal-Wallis p values analysing the relationship between trace metal concentrations and home construction materials.

**Page S13: Supplementary Table S10.** Spearman rank correlation between home age and indoor dust and garden soil trace metal concentrations.

**Page S13: Supplementary Figure S11.** Homes benchmarked for their trace metal concentrations in garden soils and indoor dust-matched locations (n = 23).

**Page S14 – S16: Supplementary Figure S12.** Spatial distribution of trace metal concentrations in garden soils and indoor dust samples from homes in the Illawarra region, represented using graduated symbol maps of various size ranges.

**Page S17: Supplementary Figure S13.** Contemporary emissions plots of various industries in the Port Kembla.

**Page S18:** **Supplementary Figure S14.** Enrichment factor calculations obtained for garden soils and indoor dust indicate the degree of “enrichment” after background normalisation.

**Page S19: Supplementary Table S15.** Descriptive statistics of enrichment factor values for garden soils and indoor dust.

**Page S20 – S23: Supplementary Table S16.** Statistical results for Hazard Quotient (HQ) and Hazard Index (HI) modelled values, representing non-carcinogenic health risks (NCHR) by exposure to trace metals in garden soil for children.

**Page S24 – S25: Supplementary Table S17.** Statistical results for carcinogenic risk (CR) modelled values, by exposure to trace metals in garden soils for children and adults.


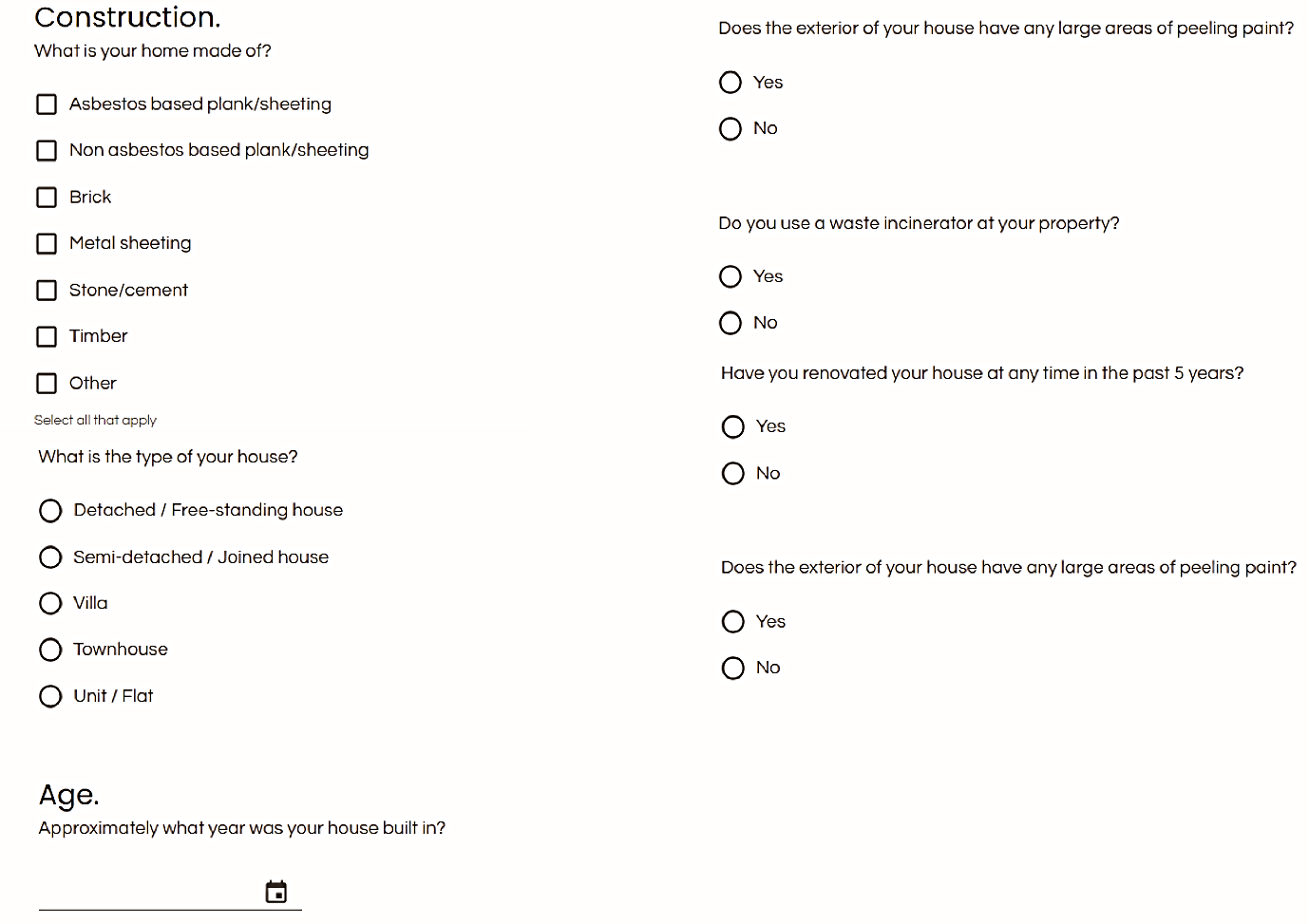


**Supplementary Figure S1.** *VegeSafe* questionnaire filled out by participants before submitting their samples to Macquarie University, detailing their home characteristics.


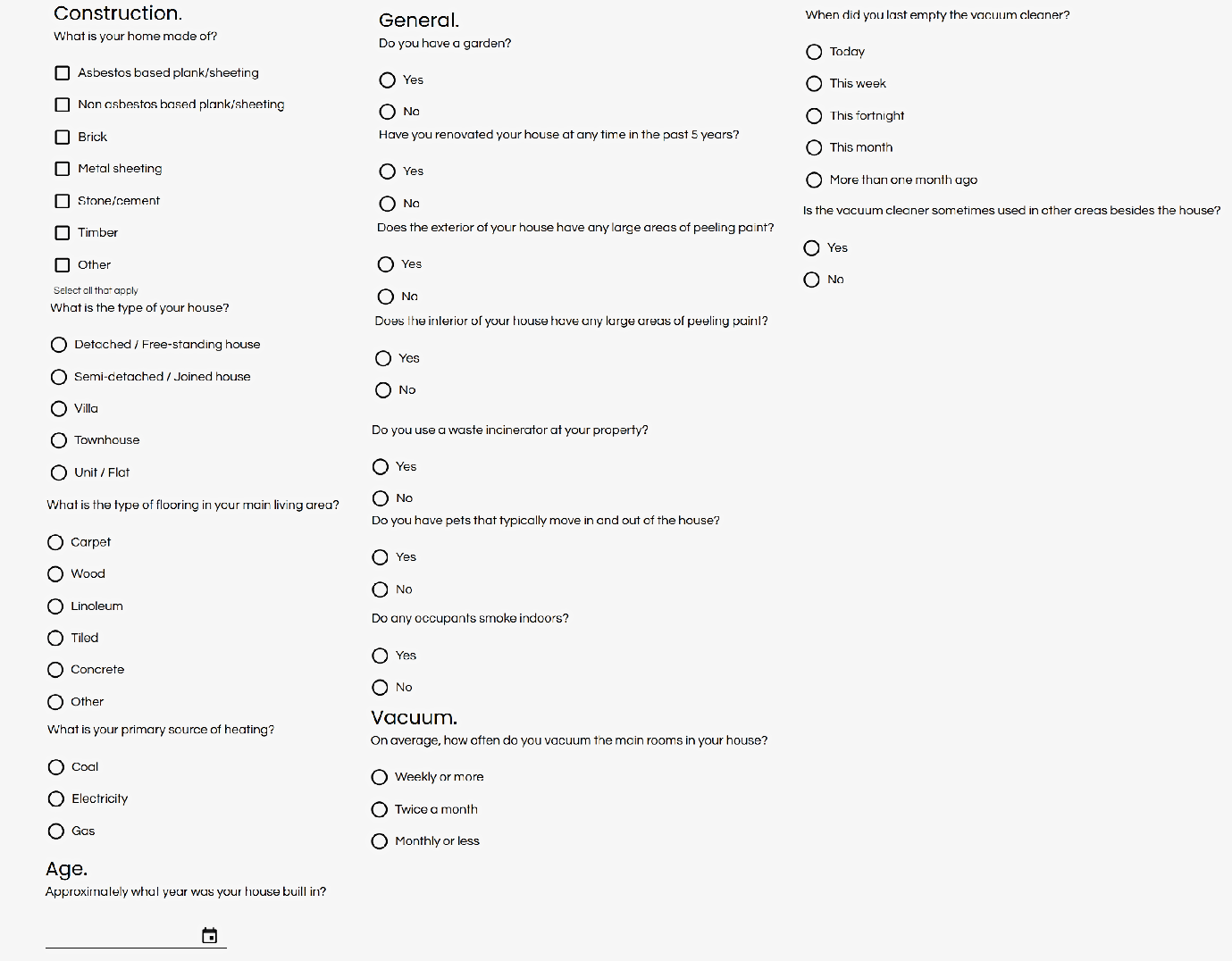
**Supplementary Figure S1.** *DustSafe* questionnaire filled out by participants before submitting their samples to Macquarie University, detailing their home characteristics.

**Supplementary Figure S2.** Report *DustSafe* participants receive outlining the concentration of trace metals in their home environment.


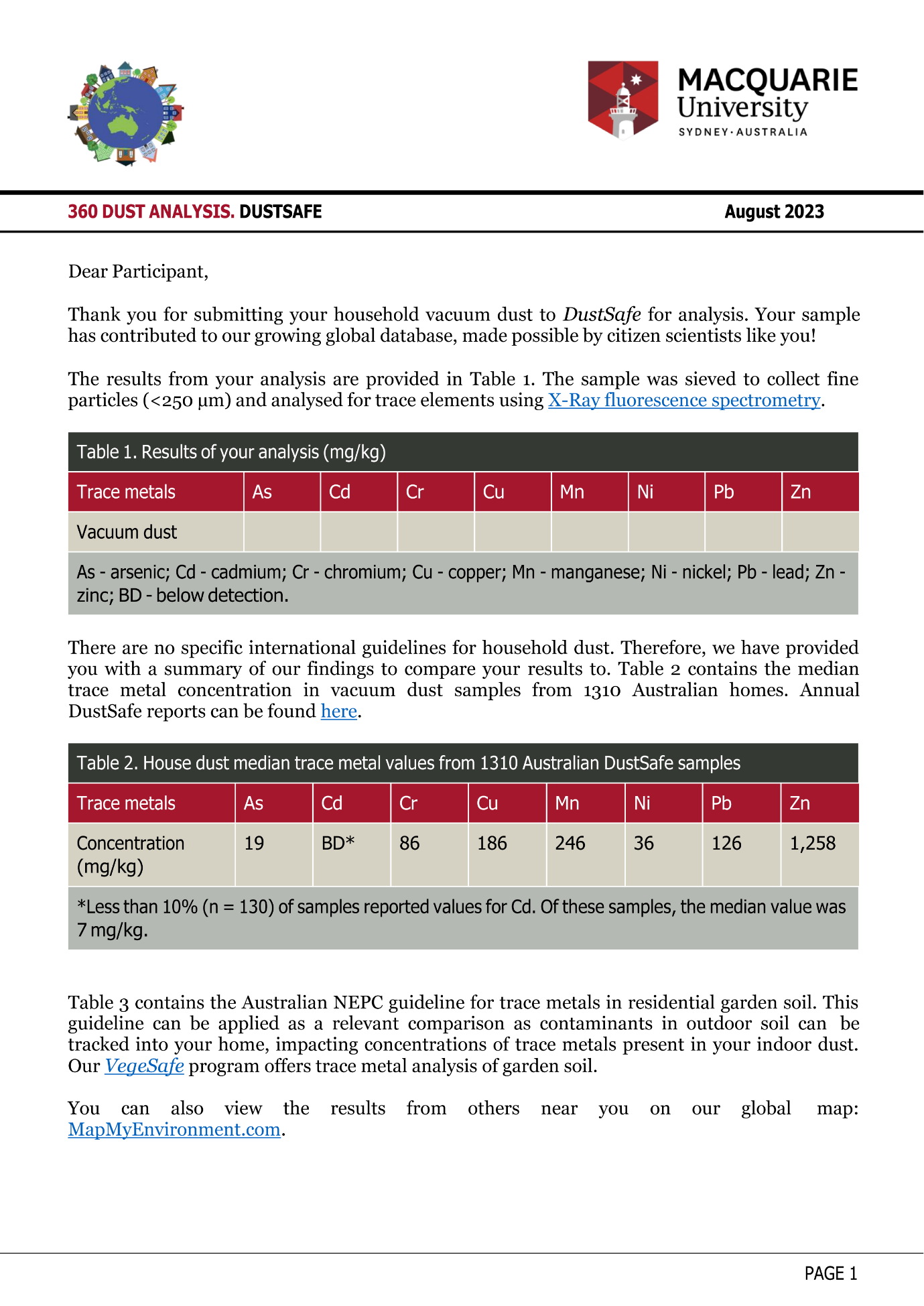

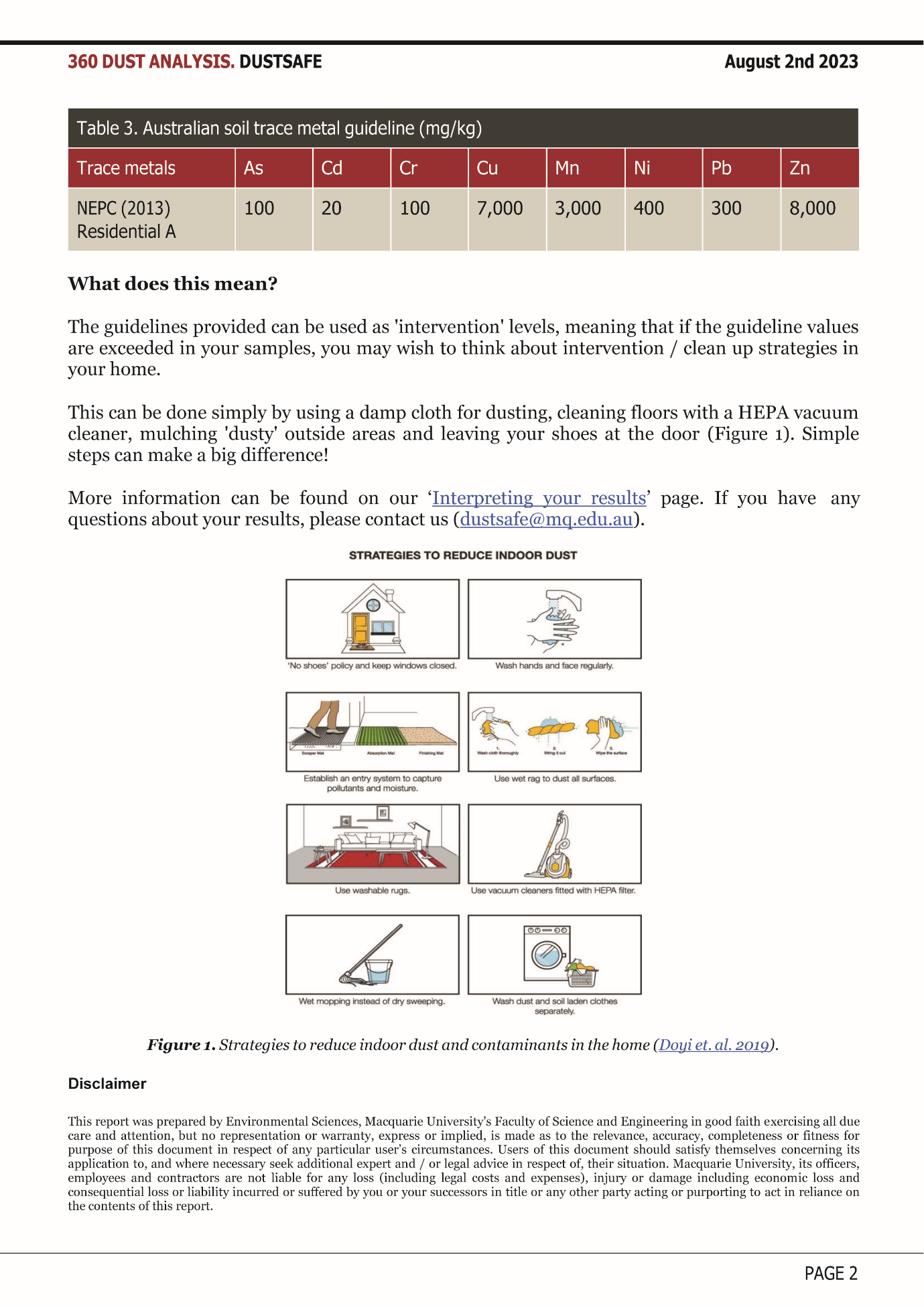


**Supplementary Figure S3.** Report *VegeSafe* participants receive outlining the concentration of trace metals in their home environment.


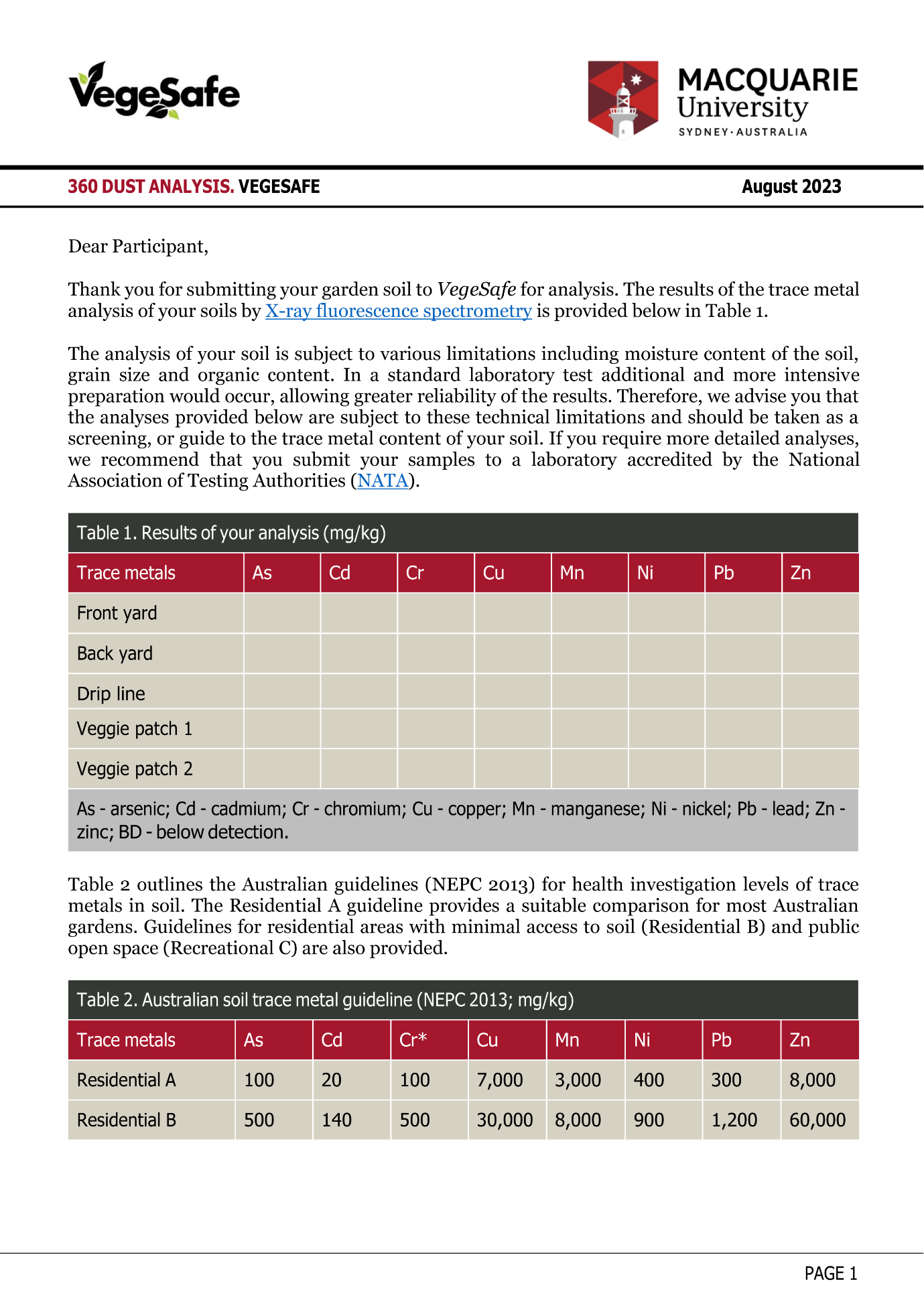

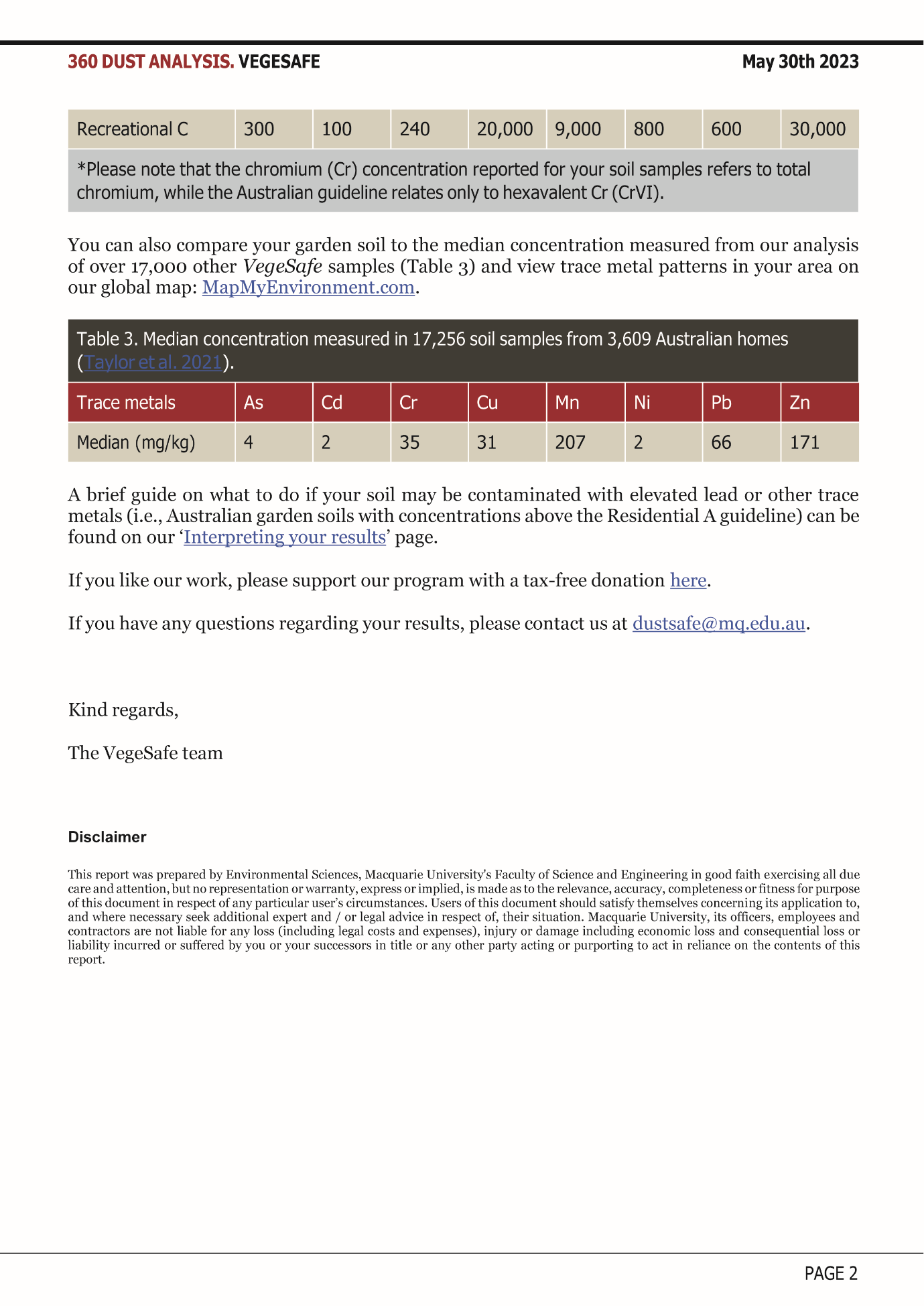


**Supplementary Table S4.** Concentrations of the six soil profiles from rural areas to determine the background of the study area. The average of the deepest horizon from the six profile locations was utilized for the calculation of EF (enrichment factor).

| **Location** | **Type of soil^a,b^** | **Geological description^c,d^** | **Sampled depth** | **As** | **Cr** | **Cu** | **Mn** | **Ni** | **Pb** | **Zn** |
| --- | --- | --- | --- | --- | --- | --- | --- | --- | --- | --- |
| Site 1  Bully | Brown Podzolic^1^ | Lithic sandstone | 0-2 | 13 | 46 | 60 | 541 | 11 | 58 | 123 |
|  |  |  | 2-10 | 13 | 50 | 64 | 485 | 9 | 57 | 123 |
|  |  |  | 10-20 | 12 | 48 | 67 | 436 | 10 | 56 | 133 |
|  |  |  | 20-30 | 11 | 54 | 61 | 421 | 9 | 57 | 129 |
|  |  |  | 30-40 | 13 | 58 | 55 | 429 | 10 | 57 | 120 |
| Site 2  Corrimal | Brown Podzolic^1^ | Lithic sandstone/ Carbonaceous claystone and coal | 0-2 | 7 | 56 | 41 | 514 | 12 | 34 | 97 |
|  |  |  | 2-10 | 6 | 68 | 40 | 513 | 11 | 34 | 99 |
|  |  |  | 10-20 | 7 | 65 | 39 | 520 | 10 | 36 | 95 |
|  |  |  | 20-30 | 6 | 80 | 40 | 498 | 9 | 32 | 97 |
|  |  |  | 30-40 | 7 | 75 | 37 | 477 | 11 | 32 | 91 |
| Site 3  Figtree | Brown Podzolic^1^ | Lithic sandstone/ Carbonaceous claystone and coal | 0-2 | 8 | 51 | 45 | 510 | 5 | 32 | 199 |
|  |  |  | 2-10 | 9 | 65 | 43 | 515 | 6 | 34 | 195 |
|  |  |  | 10-20 | 8 | 63 | 46 | 505 | 5 | 35 | 197 |
|  |  |  | 20-30 | 8 | 55 | 41 | 512 | 4 | 33 | 195 |
|  |  |  | 30-40 | 8 | 64 | 42 | 500 | 4 | 34 | 198 |
| Site 4  Dapto | Podzolic^2^ | Latite (Berkeley member) | 0-2 | 13 | 43 | 55 | 520 | 4 | 42 | 389 |
|  |  |  | 2-10 | 12 | 51 | 52 | 548 | 3 | 44 | 395 |
|  |  |  | 10-20 | 13 | 53 | 56 | 550 | 5 | 45 | 387 |
|  |  |  | 20-30 | 12 | 53 | 51 | 515 | 4 | 43 | 377 |
|  |  |  | 30-40 | 13 | 58 | 53 | 545 | 2 | 41 | 378 |
| Site 5  Penrose | Podzolic^2^ | Quartz and lithic sand, silt and clay | 0-2 | 8 | 53 | 123 | 180 | 4 | 65 | 79 |
|  |  |  | 2-10 | 7 | 67 | 125 | 195 | 4 | 63 | 65 |
|  |  |  | 10-20 | 6 | 65 | 117 | 175 | 2 | 60 | 67 |
|  |  |  | 20-30 | 8 | 57 | 116 | 171 | 5 | 63 | 57 |
|  |  |  | 30-40 | 8 | 49 | 118 | 161 | 2 | 61 | 46 |
| Site 6  Flinders | Red Podzolic^3^/Prairie^4^ | Latite-derived soil | 0-2 | 7 | 63 | 78 | 560 | 3 | 50 | 369 |
|  |  |  | 2-10 | 7 | 62 | 75 | 562 | 4 | 48 | 355 |
|  |  |  | 10-20 | 7 | 61 | 79 | 558 | 3 | 46 | 275 |
|  |  |  | 20-30 | 8 | 54 | 74 | 558 | 2 | 47 | 279 |
|  |  |  | 30-40 | 10 | 55 | 73 | 557 | 2 | 45 | 264 |
| Background mean |  |  | 0-2 | 9 | 52 | 67 | 471 | 7 | 47 | 209 |
|  |  |  | 2-10 | 9 | 61 | 67 | 470 | 6 | 47 | 205 |
|  |  |  | 10-20 | 9 | 59 | 67 | 457 | 6 | 46 | 192 |
|  |  |  | 20-30 | 9 | 59 | 64 | 446 | 6 | 46 | 189 |
|  |  |  | 30-40 | 10 | 60 | 63 | 445 | 5 | 45 | 183 |

**Sources: a) SEED (2022), b)** **Isbell (2016), c) DIGS (2022b), d) DIGS (2022a)**

^1^Brown Podzolic: derived from quartz-rich sands or sedimentary debris from igneous rocks.

^2^Podzolic: comprised of quartz sand.

^3^Red Podzolic: accumulation of compounds of organic matter, aluminium and/or iron.

^4^Prairie soils: carbon and organic matter-rich.

**Supplementary Table S5.** Detailed quality control of SRMs and sample replicates. The SRMs used were NIST2709 (San Joaquin Soil), NIST2711Montana II soil (moderately contaminated soil), NIST2583 (Trace elements in indoor dust) and NIST2584 (Trace Elements in Indoor Dust). Quality control was carried out as recovery percentage, precision (RSD) and accuracy (%D) as per US EPA 6200 method (US EPA, 2007).

| **Medium** | **SRM** | **n** | **Parameters** | **As** | **Cr** | **Cu** | **Mn** | **Ni** | **Pb** | **Zn** |
| --- | --- | --- | --- | --- | --- | --- | --- | --- | --- | --- |
| Soil | NIST2709a | 8^a^ | Certified concentration mg/kg | 10.5 | 130 | 33.9 | 529 | 85 | 17.3 | 103 |
|  |  |  | Recovery % | 95 | 113 | 88 | 96 | 94 | 92 | 96 |
|  |  |  | RSD % | 7 | 9 | 7 | 1 | 3 | 6 | 2 |
|  |  |  | %D | -5 | 13 | -12 | -4 | -6 | -8 | -4 |
|  | NIST2711a | 8^a^ | Certified concentration mg/kg | 107 | 52.3 | 140 | 675 | 21.7 | 1400 | 414 |
|  |  |  | Recovery % | 100 | 117 | 96 | 87 | 85 | 97 | 102 |
|  |  |  | RSD % | 6 | 3 | 4 | 1 | 7 | 1 | 1 |
|  |  |  | %D | 0 | 17 | -4 | -13 | -15 | -3 | 2 |
| This study dust concentration range |  |  | mg/kg | 1 - 52 | 14 - 164 | 17 - 1752 | 159 - 1866 | 1 - 206 | 3 – 1644 | 4 - 9697 |
| Replicate A, n = 8^a^ |  |  | RSD % | 6 | 6 | 16 | 13 | 8 | 3 | 8 |
| Replicate B  n = 8^a^ |  |  | RSD % | 14 | 10 | 9 | 14 | 18 | 11 | 12 |
| Dust | cc | 8^b^ | Certified concentration mg/kg | 7 | 80 |  |  |  | 85.9 |  |
|  |  |  | Recovery % | 124 | 88 | N/R | N/R | N/R | 114 | NR |
|  |  |  | RSD % | 9 | 2 | 9 | 4 | 4 | 3 | 1 |
|  |  |  | %D | 18 | -12 |  |  |  | 14 |  |
|  | NIST2584 | 8^b^ | Certified concentration mg/kg | 17.4 | 135 | 320 | 370 | 90 | 9761 | 2580 |
|  |  |  | Recovery % | N/A | 91 | 105 | 108 | 101 | 85 | 127 |
|  |  |  | RSD % | N/A | 7 | 7 | 6 | 7 | 14 | 12 |
|  |  |  | %D | N/A | -9 | 5 | 8 | 1 | -15 | 20 |
| This study soils concentration range |  |  | mg/kg | 4-88 | 69 - 667 | 84 -1166 | 212 -1177 | 16 - 139 | 47 - 1364 | 587 - 2912 |
| Replicate A  n = 8^b^ |  |  | RSD % | 7 | 5 | 11 | 3 | 11 | 4 | 4 |
| Replicate B  n = 8^b^ |  |  | RSD % | 5 | 8 | 11 | 16 | 11 | 9 | 8 |
| SiO_2_ Blank |  | 16 | Concentration mg/kg | <LOD | 0.0015 | <LOD | 0.001 | <LOD | 0.0025 | <LOD |

N/R (not reported): trace metal certified value is not reported or measured for this SRM by NIST.

N/A (not applicable): not reported due to interferences between spectrums of Pb L- and As K (USEPA, 2007).

<LOD: Limit of detection.

^a^Olympus delta VMW (tungsten tube).

^b^Olympus delta VMR (rhodium tube).

**Supplementary S6.** Equations for human health risk calculations.

The NCR was approached for the three exposure routes by first calculating the average daily dose (ADD) using the methods established by the United States Environmental Protection Agency (USEPA, 2011) as follows:

$ADDingestion = \frac{C \times IngR \times EF \times ED \times CF}{BW \times AT}$ (1)

$ADDInhalation = \frac{C \times InhR \times EF \times ED}{PEF \times BW \times AT}$ (2) $ADDDermal = \frac{C \times SA \times AF \times ABS \times EF \times ED \times CF}{BW \times AT}$ (3)

The exposure factors for children and adults and toxicological parameters were taken from the National Protection Measure Health Investigation Level A (NEPM HIL-A) for an Australian risk calculation scenario with home accessible garden (Supplementary Table S9).

Once ADDs were obtained, the hazard quotient (HQ) for non-carcinogenic risk was calculated as follows:

$HQ = \frac{ADD}{RfD}$ (4)

HQ was obtained by dividing the ADD by a reference dose (RfD), defined as the toxicity value specified for the chronic exposition to a substance of interest (Supplementary Table S8).

The Hazard Index (HI), also known as the cumulative risk, is expressed as the sum of hazard quotients from different substances (trace metals) that affect individuals through the different exposure routes (Goumenou & Tsatsakis, 2019). An HI <1.0 indicates no significant risk of adverse health effects. However, an HI > 1.0 means that “non-carcinogenic risks” are expected, and further study should be considered (NEPC, 2011).

$HI =\sum HQ=HQingestion+HQinhalation+HQdermal$ (6)

Finally, carcinogenic risk (CR) estimates a lifetime excess cancer risk that may occur in a population as a result of exposure to a particular substance (Taloor et al., 2023). The probability of developing cancer during a lifetime is calculated as the sum of individual risk cancer calculation (per trace metal) using the following equation:

$Carcinogenic risk (CR)=\sum_{k=1}^{X} ADD\times CSF$ (7)

Where the equation involves summing up each “ADD × CSF” value from the first (k = 1) to the “X” number of carcinogenic substances considered in the analysis.

Cancer slope factors (CSF) for calculations of carcinogenic risk are described in Supplementary Table S8. A calculated CR value of >1×10-4 (one case in a ten thousand population) as a result of exposure to a specific substance has been widely accepted as an unacceptable risk, values between 1×10-4 and 1×10-6 are risks of concern, and values <1×10-6 are regarded as an Acceptable Risk Level (ARL) for carcinogens (Ju et al., 2023; Petit et al., 2022).

**Supplementary Table S7.** Reference doses (RfD) and Cancer Slope Factors (CSF) for the risk calculation of the various trace metals studied. Studied trace metals such as Cr are not included because the toxicological parameters are based on CrVI. As and Pb are the only trace metals of those studied for which a CSF is available in the published literature.

| **Non-carcinogenic risk - Reference Dose (mg/kg/d)** | | | | |
| --- | --- | --- | --- | --- |
|  |  | **Ingestion** | **Inhalation** | **Dermal** |
| Reference dose  (RfD)  mg kg^-1^day^-1^ | As | 3.00×10^-4 (b, d)^ | 3.00×10^-4 (c)^ | 1.23×10^-4 (d)^ |
|  | Cu | 4.00×10^-2^ ^(a, c, e)^ | 6.9×10^-4^ ^(b)^ | 1.20×10^-2 (a, e)^ |
|  | Mn | 1.40×10^-1^ ^(b)^ | 5×10^-5 (b)^ | 5.60×10^-3 (b)^ |
|  | Ni | 2.00×10^-2 (g)^ | 9.00×10^-5 (g)^ | 5.4×10^-3 (a. d)^ |
|  | Pb | 3.5×10^-3^ ^(b, d)^ | 3.52×10^-3^ ^(c)^ | 5.30×10^-4^ ^(c)^ |
|  | Zn | 3.00×10^-1^ ^(b)^ | 3.00×10^-1 (a, c, d)^ | 6×10^-2 (a,e)^ |
| Cancer Slope Factor (CSF) | As | 1.50×10^0 (b, c)^ | 1.51×10^1 (e, h)^ | 3.66×10^0 (e)^ |
|  | Pb | 8.50×10^-3 (c, e)^ | 4.2×10^-2 (c, e)^ | - |

RfD values for non-carcinogenic and slope factors for carcinogenic risk calculations were taken from a) Aendo et al. (2022) b) IRIS (1985), c) Doyi et al. (2019), d) Hernández-Mena et al. (2021), e) Li et al. (2013), f) OEHHA (1999), g) Fay (2005).

**Supplementary Table S8.** Toxicological parameters and exposure factors for risk calculations (as per equations 1 - 3) presented as ranges in the Monte Carlo probabilistic distribution to comprehensively explore a broader parameter space. The toxicological parameters are based on the *Australian risk assessment framework* established by National Environment Council (NEPC, 2011) and The Environmental Health Standing Committee (enHealth, 2012). Distribution types were assigned for the different parameters as per (Panqing et al., 2023; Yang et al., 2019).

| **Risk calculation units and parameter** | | | | | | |
| --- | --- | --- | --- | --- | --- | --- |
| **Parameter** | **Description** | **Units** | **Child (1-2 years)** | **Adult** | **Distribution type** | **Data source** |
| ADD | Average daily dose | mg kg^-1^day^-1^ | - | - | - | Calculated (this study) |
| C | Trace metal concentration | mg kg^-1^ | - | - | Lognormal | Measured data (this study) |
| IngR | Ingestion rate | mg day^-1^ | 50 – 100 | 50 – 60 | Uniform | NEPC (2011); enHealth (2012) |
| InhR | Inhalation rate | m^3^ day^-1^ | 8 – 12.8 | 15 – 20 | Uniform | NEPC (2011); enHealth (2012) |
| EF | Exposure frequency | days year^-1^ | 350 – 365 | 350 – 365 | Uniform | NEPC (2011); enHealth (2012) |
| ED | Exposure duration | Years | 0 – 2 | 24 – 34 | Uniform | NEPC (2011); enHealth (2012) |
| BW | Body Weight | Kg | 8 – 13 | 75 – 104 | Uniform | NEPC (2011); enHealth (2012) |
| AT | Averaging time | Days | ED × 365 | ED × 365 | Uniform | NEPC (2011); enHealth (2012) |
| PEF | Particle emission factor | m^3^ kg^−1^ | 1.36 × 10^9^ | 1.36 × 10^9^ | Uniform | USEPA (1996); NEPC (2011) |
| SA | Exposed dermal area | cm^2^ | 1600 – 1900 | 20000 – 24000 | Uniform | NEPC (2011); enHealth (2012) |
| AF | Adherence factor | mg cm^-2^ | 0.5 | 0.5 | Point | USEPA (2004); NEPC (2011) |
| ABS | Dermal absorption factor | Unitless | 0.001 | 0.001 | Point | USEPA (2004); NEPC (2011) |
| CF | Conversion factor (mg to kg) |  | 1.10 × 10^-6^ | | Point | USEPA (2011) |
| RfD | Reference dose | mg kg^-1^day^-1^ | See Table 1 | | Point | See Supplementary Table S7 |
| CSF | Cancer Slope Factor | mg kg^-1^day^-1^ |  |  | Point | See Supplementary Table S7 |

**Supplementary Table S9.** Kruskal-Wallis p values analysing the relationship between trace metal concentrations and home construction materials. The construction materials brick, fibro, timber, and weatherboard did not present a significant correlation when performing Multiple Dunns tests against trace metal concentrations.

|  | As | Cr | Cu | Mn | Ni | Pb | Zn |
| --- | --- | --- | --- | --- | --- | --- | --- |
| Indoor dust (n = 23)^a^ | 0.866 | 0.473 | 0.625 | 0.642 | 0.225 | 0.303 | 0.564 |
| Garden soil n = 356 (75 homes)^b^ | 0.348 | 0.084 | 0.811 | 0.226 | 0.451 | 0.199 | 0.580 |

^a^ Materials tested were: brick n = 35, fibro n = 11, timber = 20, weatherboard = 6, other n = 3

^b^ Materials tested were: brick n = 8, fibro n = 5, timber = 5, weatherboard = 2, other n = 3

**Supplementary Table S10.** Spearman rank correlation between home age and indoor dust and garden soil trace metal concentrations. The bold p values are significant at the p < 0.05 level, and the strength of the correlation (ρ) is deemed weak if spanning ±0.1 – 0.3, moderate ±0.4 – ±0.6, strong ±0.7 – 0.9 and perfect ±1.0 (Dancey & Reidy, 2007).

|  |  | Home age |  |  | Home age |
| --- | --- | --- | --- | --- | --- |
| Indoor dust | As | **0.573** | Garden soil | As | 0.154 |
|  | Cr | 0.226 |  | Cr | 0.156 |
|  | Cu | 0.140 |  | Cu | -0.480 |
|  | Mn | 0.104 |  | Mn | -0.174 |
|  | Ni | 0.264 |  | Ni | 0.094 |
|  | Pb | **0.471** |  | Pb | -0.059 |
|  | Zn | **0.533** |  | Zn | -0.126 |


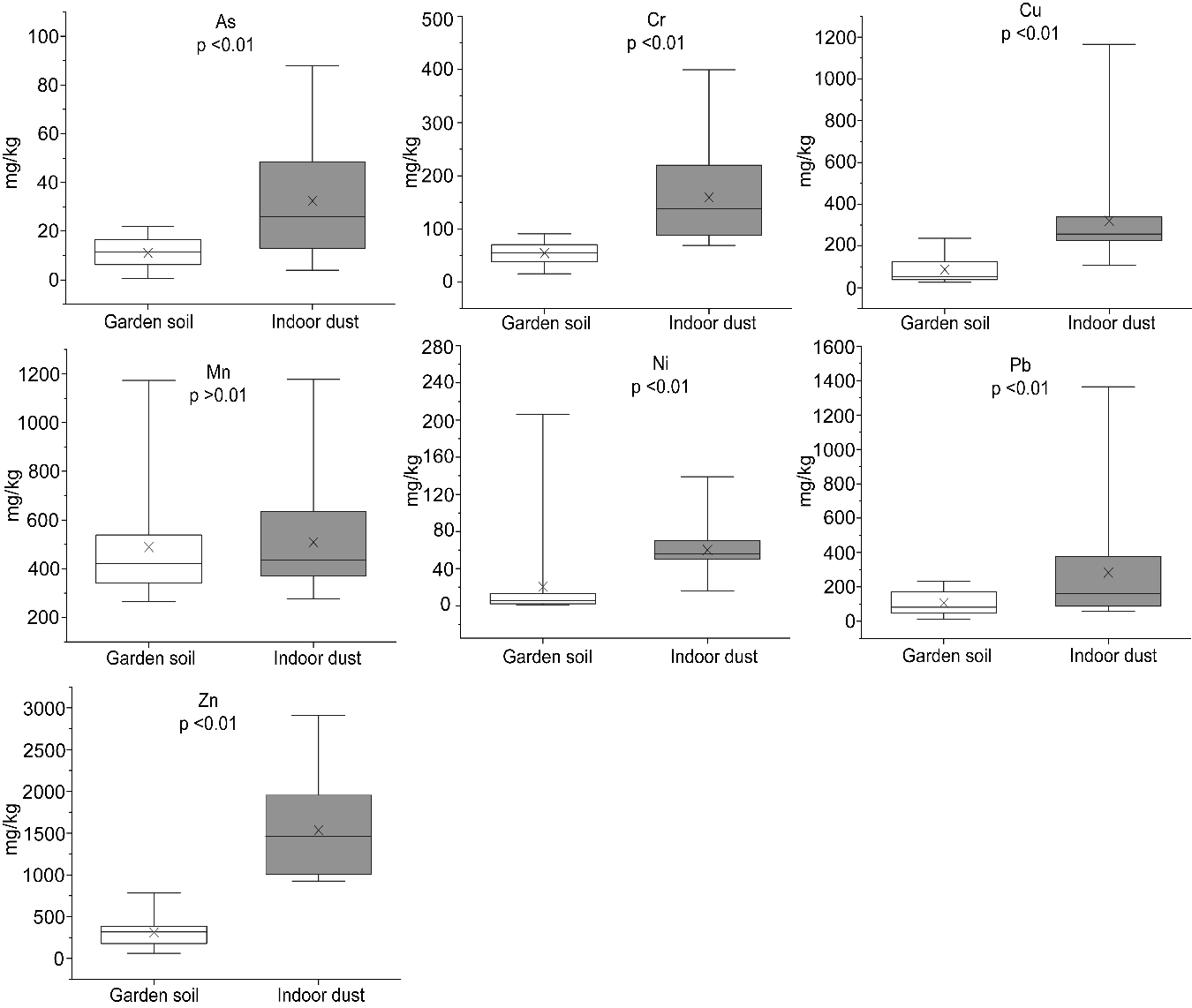


**Supplementary Figure S11.** Homes benchmarked for their trace metal concentrations in garden soils and indoor dust-matched locations (n = 23). The difference between the distribution of both media was tested at the p<0.01 level; a p >0.01 indicates no significant difference between the distribution of the two groups.

**
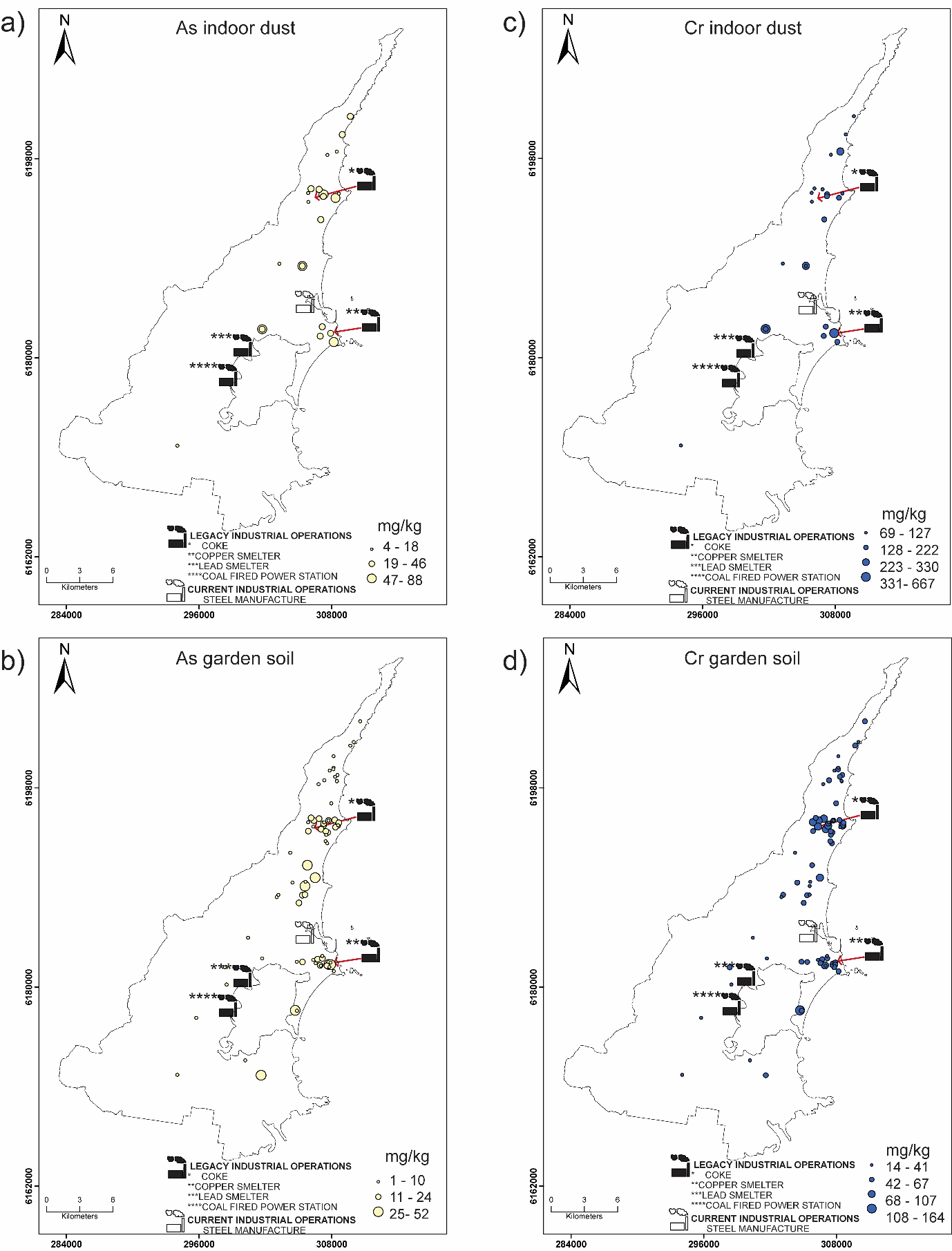
**

**Supplementary Figure S12.** Spatial distribution of trace metal concentrations in garden soils and indoor dust samples from homes in the Illawarra region, represented using graduated symbol maps of various size ranges.

**
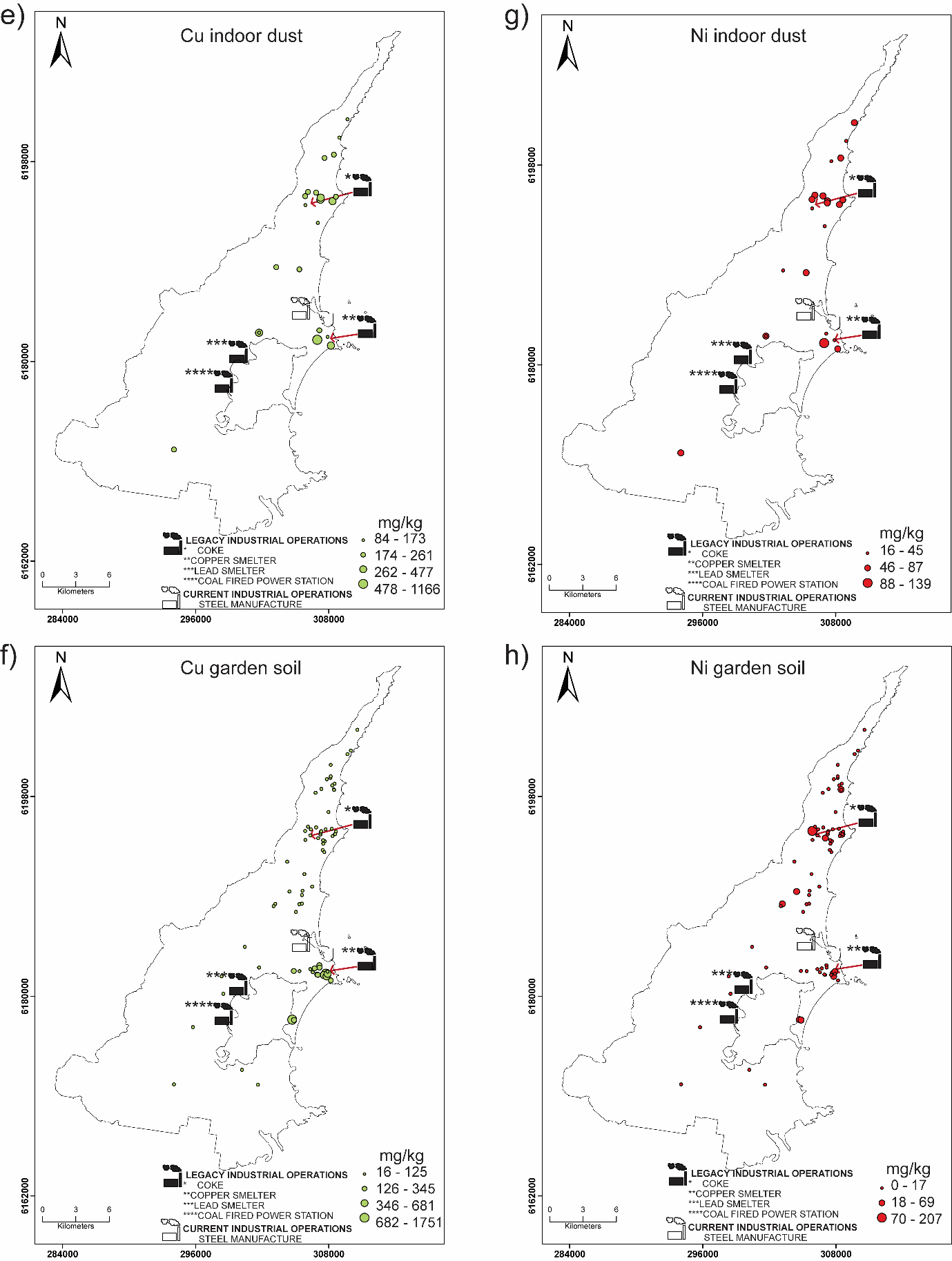
Supplementary Figure S12 continued.**


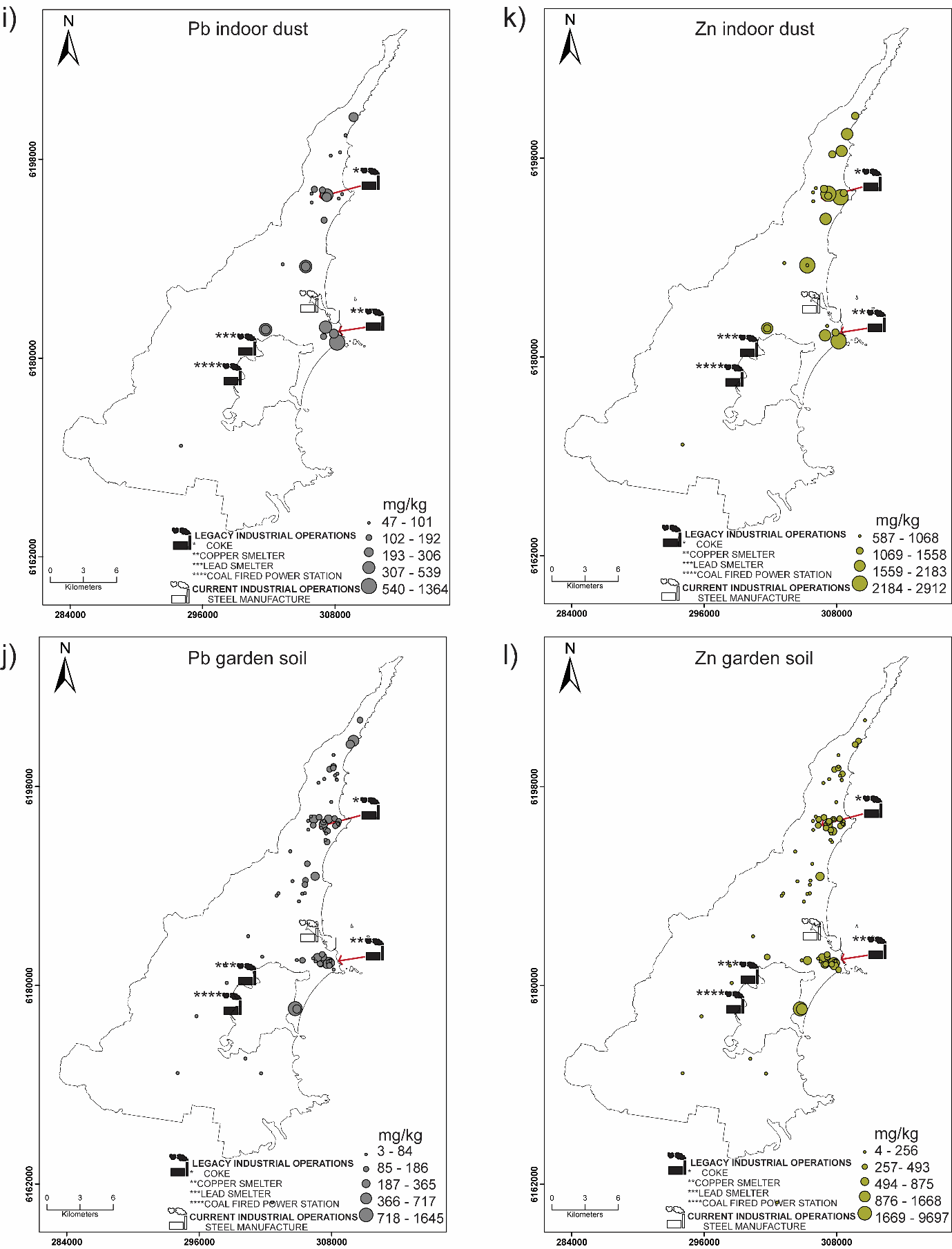
**Supplementary Figure S12 Continued.**


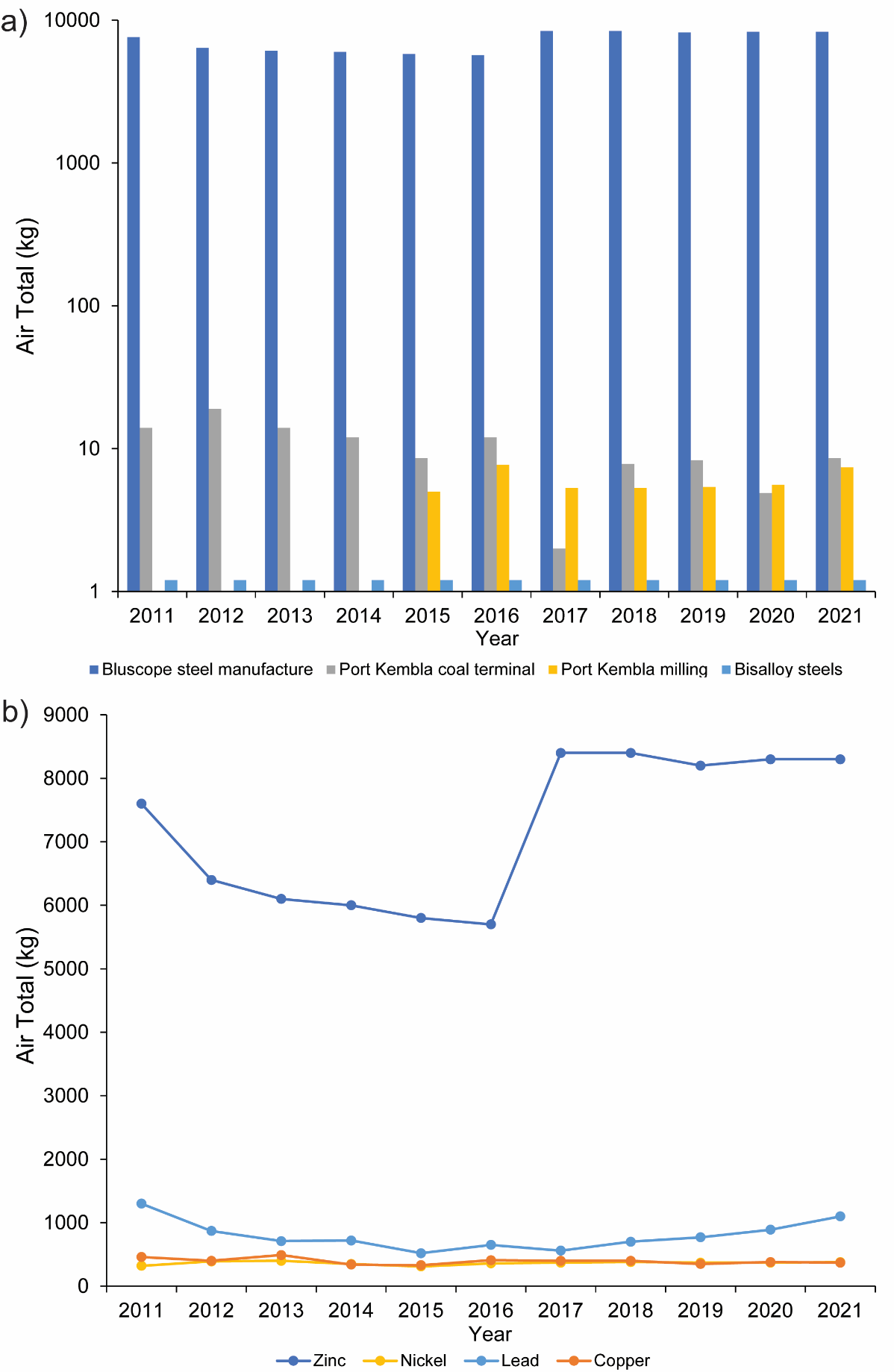


**Supplementary Figure S13.** Contemporary emissions plots of various industries in the Port Kembla area show (a) a comparison of the most significant Zn-emitting industries in the area, displaying the prominence of BlueScope steel manufacture emissions compared to Port Kembla coal terminal, Port Kembla milling, and Bisalloy steels. The comparison of air emissions in BlueScope steel (b) shows significant Zn emissions compared to other trace metals (Ni, Pb and Cu).


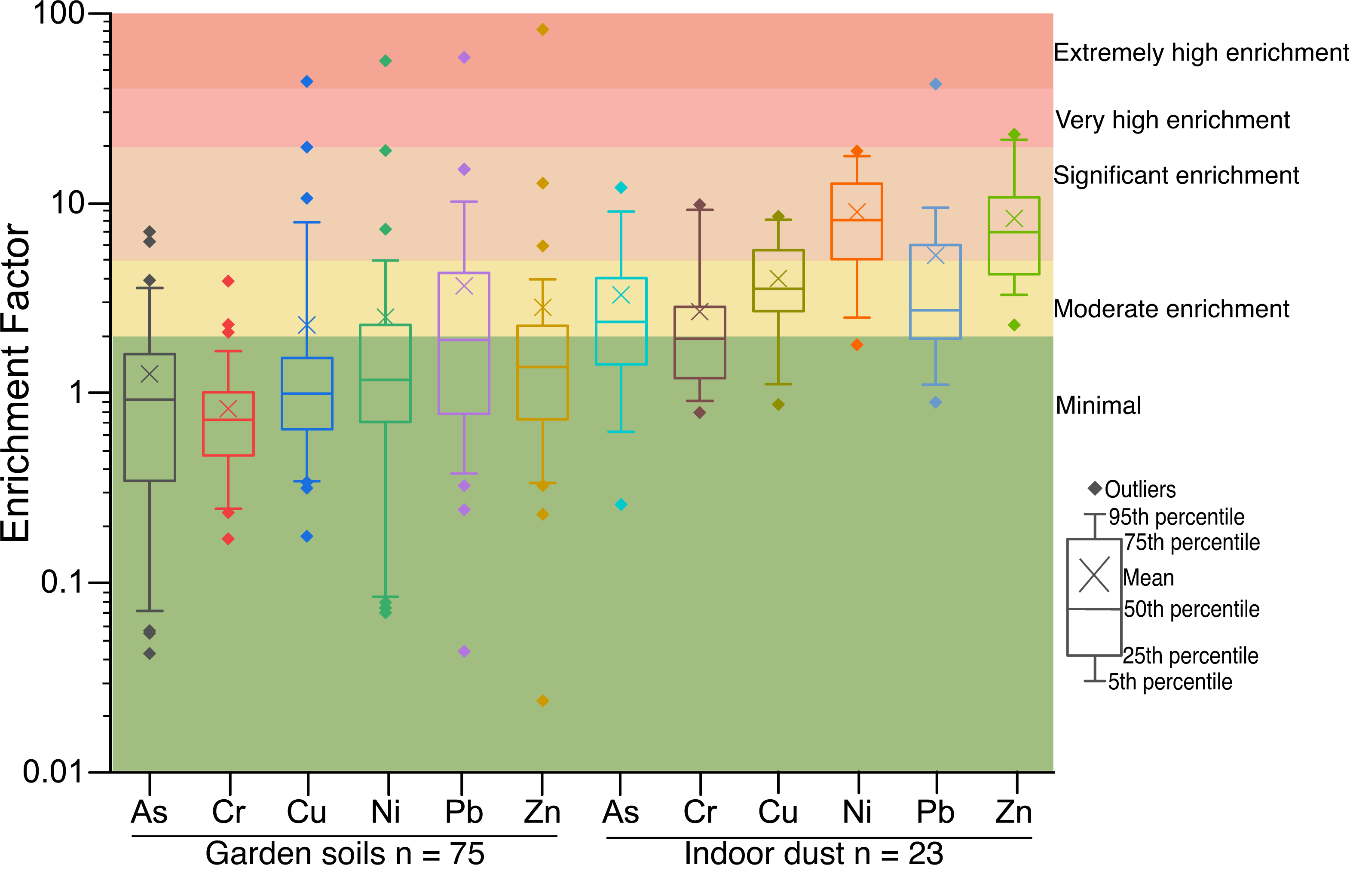
 **Supplementary Figure S14.** Enrichment factor calculations obtained for garden soils and indoor dust indicate the degree of “enrichment” after background normalisation.

**Supplementary Table S15.** Descriptive statistics of enrichment factor values for garden soils and indoor dust. EF classification details the percentage of locations enriched within the corresponding group. Reported mean EF values for Sydney were taken from Ibañez-Del Rivero et al. (2023) and for Noumea were extracted from Fry et al. (2021).

|  | **Garden soil (n = 75)** | | | | | | |
| --- | --- | --- | --- | --- | --- | --- | --- |
|  | | **As** | **Cr** | **Cu** | **Ni** | **Pb** | **Zn** |
| Min | | 0.04 | 0.17 | 0.18 | 0.07 | 0.04 | 0.02 |
| 5 | | 0.10 | 0.27 | 0.34 | 0.09 | 0.39 | 0.38 |
| 25 | | 0.35 | 0.47 | 0.65 | 0.71 | 0.79 | 0.73 |
| 50 | | 0.92 | 0.72 | 0.99 | 1.17 | 1.90 | 1.37 |
| 75 | | 1.59 | 1.00 | 1.53 | 2.26 | 3.78 | 2.23 |
| 95 | | 3.58 | 1.58 | 7.85 | 4.44 | 8.81 | 3.96 |
| Max | | 7.07 | 3.88 | 43.95 | 56.43 | 58.70 | 82.30 |
| Mean | | 1.23 | 0.83 | 2.28 | 2.50 | 3.68 | 2.83 |
| **EF classification** | | % (n) | % | % | % | % | % |
| Minimal enrichment | | 81 | 96 | 87 | 69 | 51 | 72 |
| Moderate enrichment | | 16 | 4 | 5 | 26 | 32 | 24 |
| Significant enrichment | | 3 |  | 7 | 4 | 16 | 3 |
| Very High enrichment | |  |  |  |  |  |  |
| Extremely High enrichment | |  |  | 1 | 1 | 1 | 1 |
|  | **Indoor dust (n = 23)** | | | | | | |
|  | | **As** | **Cr** | **Cu** | **Ni** | **Pb** | **Zn** |
| Min | | 0.26 | 0.79 | 0.87 | 1.79 | 0.89 | 2.29 |
| 5 | | 0.65 | 0.91 | 1.14 | 2.52 | 1.11 | 3.30 |
| 25 | | 1.42 | 1.22 | 2.71 | 5.81 | 1.94 | 4.23 |
| 50 | | 2.37 | 1.93 | 3.54 | 8.14 | 2.72 | 7.04 |
| 75 | | 3.69 | 2.79 | 5.45 | 12.50 | 5.40 | 10.09 |
| 95 | | 9.03 | 8.80 | 8.04 | 17.41 | 9.32 | 20.92 |
| Max | | 12.09 | 9.84 | 8.54 | 18.86 | 42.46 | 23.10 |
| Mean | | 3.28 | 2.68 | 4.00 | 8.99 | 5.32 | 8.32 |
| **EF classification** | | % | % | % | % | % | % |
| Minimal enrichment | | 44 | 52 | 13 | 4 | 31 |  |
| Moderate enrichment | | 39 | 39 | 57 | 17 | 43 | 34 |
| Significant enrichment | | 17 | 9 | 30 | 79 | 22 | 57 |
| Very High enrichment | |  |  |  |  |  | 9 |
| Extremely High enrichment | |  |  |  |  | 4 |  |
|  | |  |  |  |  |  |  |
|  | | Sydney | | | | | |
|  | | **As** | **Cr** | **Cu** | **Ni** | **Pb** | **Zn** |
| EF garden soils mean (n = 166) | | 7 | 2 | 8 | - | 53 | 16 |
| EF indoor dusts mean (n = 166) | | 12 | 3 | 22 | - | 42 | 53 |
|  | | Noumea | | | | | |
|  | | **As** | **Cr** | **Cu** | **Ni** | **Pb** | **Zn** |
| EF garden soils mean (n = 15) | | 1 | 5 | 1 | 6 | 3 | 4 |
| EF indoor dusts mean (n = 39) | | 2 | 8 | 9 | 16 | 26 | 48 |

**Supplementary Table S16.** Statistical results for Hazard Quotient (HQ) and Hazard Index (HI) modelled values, representing non-carcinogenic health risks (NCHR) by exposure to trace metals in garden soil for children. These results were derived from a probabilistic risk assessment encompassing 10,000 iterations for home garden soils in the Illawarra region and garden soils across Australian homes.

|  | **NCHR modelled in home garden soil for children** | | | | | | | | | | | | |
| --- | --- | --- | --- | --- | --- | --- | --- | --- | --- | --- | --- | --- | --- |
|  |  | **Illawarra region homes** | | | | | | **Australian homes** | | | | | |
|  |  | **Mean** | **5%** | **Median** | **95%** | **Minimum** | **Maximum** | **Mean** | **5%** | **Median** | **95%** | **Minimum** | **Maximum** |
| **HQ Dermal** | As | 5.05×10^−4^ | 1.65×10^−5^ | 2.79×10^−4^ | 1.72×10^−3^ | 2.50×10^−10^ | 1.05×10^−2^ | 5.09×10^−4^ | 2.50×10^−5^ | 1.84×10^−4^ | 2.19×10^−3^ | 9.20×10^−6^ | 5.68×10^−2^ |
|  | Cu | 3.45×10^−3^ | 1.08×10^−4^ | 2.55×10^−3^ | 9.78×10^−3^ | 1.67×10^−8^ | 1.90×10^−2^ | 3.54×10^−3^ | 4.44×10^−5^ | 1.57×10^−3^ | 1.35×10^−2^ | 4.10×10^−6^ | 1.19×10^−1^ |
|  | Mn | 7.51×10−^4^ | 2.34×10^−5^ | 5.56×10^−4^ | 2.13×10^−3^ | 3.64×10^−9^ | 4.14×10^−3^ | 5.91×10^−4^ | 1.22×10^−5^ | 2.95×10^−4^ | 2.12×10^−3^ | 1.20×10^−6^ | 1.79×10^−2^ |
|  | Ni | 5.07×10−^4^ | 1.09×10^−5^ | 1.90×10^−4^ | 1.93×10^−3^ | 2.29×10^−8^ | 3.36×10^−2^ | 4.10×10^−4^ | 9.90×10^−5^ | 1.57×10^−4^ | 2.52×10^−3^ | 1.07×10^−6^ | 1.43×10^−2^ |
|  | Pb | 1.98×10^−2^ | 1.67×10^−3^ | 1.65×10^−2^ | 3.89×10^−2^ | 1.00×10^−6^ | 6.21×10^−2^ | 2.71×10^−2^ | 1.25×10^−4^ | 5.30×10^−3^ | 1.16×10^−1^ | 1.50×10^−5^ | 6.80×10^-1^ |
|  | Zn | 5.40×10^−4^ | 2.04×10^−5^ | 2.98×10^−4^ | 1.89×10^−3^ | 6.23×10^−9^ | 1.42×10^−2^ | 4.15×10^−4^ | 4.71×10^−6^ | 1.51×10^−4^ | 1.64×10^−3^ | 2.70×10^−7^ | 2.02×10^−2^ |
|  | **Σ** | **2.56×10^−2^** |  |  |  |  |  | **3.26×10^−2^** |  |  |  |  |  |
| **HQ Ingestion** | As | 4.80×10^−2^ | 1.51×10^−3^ | 8.63×10^−3^ | 1.66×10^−2^ | 4.03×10^−7^ | 6.10×10^-2^ | 4.59×10^−2^ | 5.92×10^−4^ | 1.66×10^−2^ | 4.96×10^−2^ | 9.11×10^−5^ | 6.88×10^-2^ |
|  | Cu | 1.13×10^−2^ | 3.87×10^−4^ | 8.31×10^−3^ | 3.22×10^−2^ | 4.41×10^−8^ | 5.11×10^−2^ | 1.24×10^−2^ | 1.66×10^−4^ | 5.41×10^−3^ | 1.76×10^−2^ | 2.17×10^−5^ | 3.69×10^−2^ |
|  | Mn | 9.01×10^−3^ | 2.74×10^−4^ | 6.63×10^−3^ | 2.55×10^−2^ | 4.14×10^−8^ | 4.92×10^−2^ | 6.61×10^−3^ | 1.35×10^−4^ | 3.38×10^−3^ | 2.40×10^−2^ | 3.60×10^−5^ | 1.72×10^−1^ |
|  | Ni | 1.34×10^−3^ | 1.34×10^−5^ | 4.18×10^−4^ | 5.28×10^−3^ | 7.81×10^−9^ | 1.38×10^−1^ | 1.65×10^−3^ | 4.42×10^−4^ | 6.10×10^−4^ | 1.05×10^−2^ | 4.66×10^−5^ | 6.37×10^−2^ |
|  | Pb | 7.07×10^−2^ | 6.40×10^−3^ | 6.31×10^−2^ | 1.63×10^−1^ | 3.67×10^−6^ | 3.64×10^−1^ | 7.84×10^−2^ | 3.46×10^−4^ | 1.67×10^−2^ | 3.28×10^−1^ | 6.00×10^−4^ | 1.35×10^0^ |
|  | Zn | 9.34×10^−3^ | 3.42×10^−4^ | 4.97×10^−3^ | 3.29×10^−2^ | 1.31×10^−8^ | 3.18×10^−1^ | 8.20×10^−3^ | 1.02×10^−4^ | 3.07×10^−3^ | 3.21×10^−2^ | 8.50×10^−5^ | 3.95×10^−1^ |
|  | **Σ** | **1.39×10^−1^** |  |  |  |  |  | **1.53×10^−1^** |  |  |  |  |  |
| **HQ Inhalation** | As | 3.98×10^−4^ | 5.39×10^−6^ | 1.82×10^−4^ | 1.53×10^−3^ | 6.26×10^−8^ | 1.33×10^−2^ | 4.05×10^−4^ | 1.20×10^−7^ | 1.42×10^−4^ | 1.74×10^−3^ | 7.00×10^−8^ | 5.88×10^−2^ |
|  | Cu | 8.41×10^−4^ | 2.56×10^−5^ | 6.19×10^−4^ | 2.38×10^−3^ | 3.87×10^−9^ | 4.60×10^−3^ | 8.54×10^−4^ | 1.08×10^−5^ | 3.79×10^−4^ | 3.22×10^−3^ | 8.40×10^−6^ | 2.08×10^−2^ |
|  | Mn | 6.29×10^−6^ | 2.15×10^−7^ | 4.63×10^−6^ | 1.79×10^−5^ | 2.44×10^−11^ | 2.84×10^−5^ | 6.83×10^−6^ | 1.39×10^−7^ | 3.56×10^−6^ | 2.44×10^−5^ | 3.40×10^−8^ | 1.64×10^−3^ |
|  | Ni | 3.63×10^−5^ | 7.92×10^−7^ | 1.39×10^−5^ | 1.37×10^−4^ | 2.48×10^−9^ | 2.40×10^−3^ | 2.85×10^−5^ | 7.90×10^−9^ | 1.03×10^−5^ | 1.89×10^−4^ | 7.70×10^−10^ | 7.62×10^−4^ |
|  | Pb | 1.42×10^−2^ | 4.99×10^−4^ | 1.04×10^−2^ | 4.09×10^−2^ | 6.61×10^−7^ | 7.28×10^−2^ | 1.64×10^−2^ | 6.15×10^−5^ | 2.96×10^−3^ | 6.06×10^−2^ | 4.40×10^−9^ | 4.93×10^-1^ |
|  | Zn | 1.11×10^−4^ | 1.81×10^−6^ | 4.99×10^−5^ | 4.00×10^−4^ | 1.85×10^−9^ | 8.49×10^−3^ | 9.65×10^−5^ | 1.22×10^−6^ | 3.53×10^−5^ | 3.70×10^−4^ | 1.20×10^−10^ | 5.96×10^−3^ |
|  | **Σ** | **1.56×10^−2^** |  |  |  |  |  | **1.78×10^−2^** |  |  |  |  |  |
| **HI** | As | 4.89×10^−2^ | 1.90×10^−3^ | 2.69×10^−2^ | 1.68×10^−1^ | 4.66×10^−7^ | 6.41×10^-2^ | 4.68×10^−2^ | 3.51×10^−3^ | 1.72×10^−2^ | 1.98×10^−1^ | 2.18×10^−7^ | 1.80×10^-1^ |
|  | Cu | 1.56×10^−2^ | 2.09×10^−3^ | 1.32×10^−2^ | 3.70×10^−2^ | 8.02×10^−5^ | 6.82×10^−2^ | 1.68×10^−2^ | 8.28×10^−4^ | 9.07×10^−3^ | 5.72×10^−2^ | 3.22×10^−6^ | 2.02×10^−1^ |
|  | Mn | 9.77×10^−3^ | 3.03×10^−4^ | 7.19×10^−3^ | 2.77×10^−2^ | 2.21×10^−6^ | 5.30×10^−2^ | 7.21×10^−3^ | 1.75×10^−4^ | 3.89×10^−3^ | 2.55×10^−2^ | 4.82×10^−6^ | 2.01×10^−1^ |
|  | Ni | 1.88×10^−3^ | 6.25×10^−5^ | 7.27×10^−4^ | 6.91×10^−3^ | 6.15×10^−7^ | 3.34×10^−1^ | 2.09×10^−3^ | 2.96×10^−5^ | 8.47×10^−4^ | 1.17×10^−2^ | 6.54×10^−7^ | 8.12×10^−2^ |
|  | Pb | 1.05×10^−1^ | 1.88×10^−2^ | 9.44×10^−2^ | 2.18×10^−1^ | 3.27×10^−4^ | 4.85×10^−1^ | 1.22×10^−1^ | 3.48×10^−3^ | 4.58×10^−2^ | 4.63×10^−1^ | 7.70×10^−5^ | 2.83×10^0^ |
|  | Zn | 9.99×10^−3^ | 4.15×10^−4^ | 5.35×10^−3^ | 3.51×10^−2^ | 2.12×10^−8^ | 3.33×10^−1^ | 8.71×10^−3^ | 2.00×10^−4^ | 3.53×10^−3^ | 3.34×10^−2^ | 4.52×10^−6^ | 3.99×10^−1^ |
|  | **Σ** | **1.80×10^−1^** |  |  |  |  |  | **2.04×10^−1^** |  |  |  |  |  |

**Supplementary Table S16 continued.** Statistical results for Hazard Quotient (HQ) and Hazard Index (HI) modelled values, representing non-carcinogenic health risks (NCHR) by exposure to trace metals in garden soil for adults. These results were derived from a probabilistic risk assessment encompassing 10,000 iterations for home garden soils in the Illawarra region and garden soils across Australian homes.

|  | **NCHR modelled in home garden soil for adults** | | | | | | | | | | | | |
| --- | --- | --- | --- | --- | --- | --- | --- | --- | --- | --- | --- | --- | --- |
|  |  | **Illawarra region homes** | | | | | | **Australian homes** | | | | | |
|  |  | **Mean** | **5%** | **Median** | **95%** | **Minimum** | **Maximum** | **Mean** | **5%** | **Median** | **95%** | **Minimum** | **Maximum** |
| **HQ Dermal** | As | 1.71×10^−4^ | 3.08×10^−6^ | 7.54×10^−5^ | 6.41×10^−4^ | 1.52×10^−11^ | 5.96×10^−3^ | 1.80×10^−4^ | 2.70×10^−6^ | 8.66×10^−5^ | 7.00×10^−4^ | 2.00×10^−10^ | 2.73×10^−2^ |
|  | Cu | 2.20×10^−5^ | 4.66×10^−7^ | 8.52×10^−6^ | 8.22×10^−5^ | 3.64×10^−10^ | 1.85×10^−3^ | 2.35×10^−5^ | 7.00×10^−7^ | 1.31×10^−5^ | 8.08×10^−5^ | 3.30×10^−10^ | 5.58×10^−4^ |
|  | Mn | 1.93×10^−4^ | 7.41×10^−6^ | 1.20×10^−4^ | 6.06×10^−4^ | 1.52×10^−8^ | 3.64×10^−3^ | 1.91×10^−4^ | 1.03×10^−5^ | 1.19×10^−4^ | 6.07×10^−4^ | 7.70×10^−8^ | 3.66×10^−3^ |
|  | Ni | 4.89×10^−6^ | 6.55×10^−8^ | 1.58E×10^−6^ | 2.02×10^−5^ | 3.02×10^−11^ | 3.75×10^−4^ | 4.89×10^−6^ | 1.30×10^−5^ | 2.79×10^−6^ | 2.77×10^−5^ | 6.40×10^−7^ | 1.06×10^−4^ |
|  | Pb | 2.11×10^−2^ | 7.21×10^−4^ | 1.55×10^−2^ | 6.00×10^−2^ | 8.18×10^−8^ | 9.50×10^−2^ | 3.22×10^−2^ | 2.83×10^−4^ | 7.80×10^−3^ | 1.28×10^−1^ | 3.70×10^−7^ | 5.36×10^-1^ |
|  | Zn | 1.39×10^−3^ | 2.95×10^−5^ | 6.13×10^−4^ | 5.22×10^−3^ | 5.76×10^−9^ | 3.76×10^−2^ | 1.35×10^−3^ | 3.74×10^−5^ | 6.33×10^−4^ | 4.90×10^−3^ | 3.40×10^−7^ | 8.26×10^−2^ |
|  | **Σ** | **2.29×10^−2^** |  |  |  |  |  | **3.39×10^−2^** |  |  |  |  |  |
| **HQ Ingestion** | As | 1.04×10^−2^ | 1.19×10^−4^ | 4.58×10^−3^ | 3.93×10^−2^ | 1.13×10^−8^ | 2.27×10^−1^ | 1.17×10^−2^ | 2.76×10^−4^ | 5.51×10^−3^ | 4.62×10^−2^ | 1.32×10^−7^ | 3.12×10^−1^ |
|  | Cu | 4.45×10^−3^ | 6.68×10^−5^ | 1.73×10^−3^ | 1.70×10^−2^ | 4.55×10^−8^ | 4.24×10^−2^ | 4.52×10^−3^ | 1.44×10^−4^ | 2.46×10^−3^ | 1.53×10^−2^ | 5.40×10^−8^ | 3.67×10^−1^ |
|  | Mn | 3.86×10^−3^ | 1.51×10^−4^ | 2.42×10^−3^ | 1.23×10^−2^ | 3.04×10^−7^ | 7.44×10^−2^ | 3.24×10^−3^ | 1.53×10^−4^ | 2.00×10^−3^ | 1.04×10^−2^ | 1.40×10^−8^ | 7.65×10^−2^ |
|  | Ni | 6.63×10^−6^ | 8.86×10^−8^ | 2.13×10^−6^ | 2.76×10^−5^ | 4.26×10^−11^ | 5.30×10^−4^ | 6.15×10^−6^ | 8.50×10^−8^ | 3.48×10^−6^ | 3.38×10^−5^ | 8.40×10^−10^ | 1.26×10^−4^ |
|  | Pb | 4.40×10^−2^ | 1.37×10^−3^ | 3.26×10^−2^ | 1.25×10^−1^ | 2.13×10^−7^ | 2.43×10^−1^ | 5.29×10^−2^ | 4.48×10^−4^ | 1.32×10^−2^ | 2.16×10^−1^ | 2.00×10^−8^ | 1.05×10^0^ |
|  | Zn | 1.39×10^−3^ | 2.94×10^−5^ | 6.20×10^−4^ | 5.22×10^−3^ | 5.22×10^−9^ | 3.57×10^−2^ | 2.02×10^−3^ | 5.47×10^−5^ | 9.22×10^−4^ | 7.38×10^−3^ | 4.30×10^−10^ | 7.26×10^−2^ |
|  | **Σ** | **6.41×10^−2^** |  |  |  |  |  | **7.44×10^−2^** |  |  |  |  |  |
| **HQ Inhalation** | As | 4.49×10^−4^ | 8.26×10^−6^ | 1.98×10^−4^ | 1.67×10^−3^ | 4.58×10^−9^ | 1.67×10^−2^ | 5.10×10^−4^ | 9.40×10^−6^ | 1.75×10^−4^ | 2.22×10^−3^ | 8.70×10^−9^ | 1.22×10^−2^ |
|  | Cu | 3.31×10^−5^ | 6.94×10^−7^ | 1.28×10^−5^ | 1.24×10^−4^ | 5.46×10^−10^ | 2.79×10^−3^ | 3.29×10^−5^ | 4.32×10^−7^ | 1.44×10^−5^ | 1.24×10^−4^ | 2.60×10^−10^ | 6.99×10^−3^ |
|  | Mn | 2.76×10^−7^ | 5.78×10^−9^ | 1.06×10^−7^ | 1.03×10^−6^ | 4.55×10^−12^ | 2.33×10^−5^ | 3.79×10^−7^ | 7.23×10^−9^ | 1.92×10^−7^ | 1.37×10^−6^ | 3.40×10^−12^ | 8.51×10^−5^ |
|  | Ni | 2.52×10^−5^ | 6.86×10^−7^ | 1.56E×10^−5^ | 8.31×10^−5^ | 1.51×10^−10^ | 4.76×10^−4^ | 2.17×10^−5^ | 4.60×10^−7^ | 6.83×10^−6^ | 1.39×10^−4^ | 3.50×10^−10^ | 7.83×10^−4^ |
|  | Pb | 1.02×10^−2^ | 3.11×10^−4^ | 7.51×10^−3^ | 2.89×10^−2^ | 4.69×10^−8^ | 5.58×10^−2^ | 1.00×10^−2^ | 3.74×10^−6^ | 1.84×10^−3^ | 3.95×10^−2^ | 1.10×10^−9^ | 3.54×10^-1^ |
|  | Zn | 6.05×10^−4^ | 8.61×10^−6^ | 2.64×10^−4^ | 2.32×10^−3^ | 9.71×10^−10^ | 1.73×10^−2^ | 6.05×10^−4^ | 6.66×10^−8^ | 2.22×10^−4^ | 2.53×10^−3^ | 2.50×10^−10^ | 2.36×10^−2^ |
|  | **Σ** | **1.47×10^−2^** |  |  |  |  |  | **1.12×10^−2^** |  |  |  |  |  |
| **HI** | As | 1.10×10^−2^ | 3.93×10^−4^ | 5.32×10^−3^ | 3.97×10^−2^ | 1.52×10^−11^ | 2.28×10^−1^ | 1.24×10^−2^ | 8.38×10^−3^ | 6.14×10^−3^ | 4.75×10^−2^ | 9.31×10^−4^ | 3.65×10^−1^ |
|  | Cu | 4.47×10^−3^ | 7.84×10^−5^ | 1.74×10^−3^ | 1.70×10^−2^ | 1.03×10^−6^ | 4.24×10^−2^ | 4.57×10^−3^ | 1.81×10^−4^ | 2.51×10^−3^ | 1.54×10^−2^ | 9.23×10^−5^ | 3.67×10^−1^ |
|  | Mn | 4.08×10^−3^ | 1.77×10^−4^ | 2.56×10^−3^ | 1.29×10^−2^ | 2.29×10^−6^ | 7.78×10^−2^ | 3.43×10^−3^ | 1.91×10^−4^ | 2.19×10^−3^ | 1.08×10^−2^ | 6.87×10^−7^ | 8.10×10^−2^ |
|  | Ni | 3.40×10^−3^ | 3.97×10^−5^ | 1.09×10^−3^ | 1.41×10^−2^ | 7.80×10^−8^ | 1.55×10^−1^ | 3.27×10^−5^ | 8.12×10^−6^ | 2.06×10^−5^ | 1.56×10^−4^ | 8.33×10^−7^ | 1.03×10^−3^ |
|  | Pb | 7.53×10^−2^ | 1.05×10^−2^ | 6.38×10^−2^ | 1.80×10^−1^ | 2.04×10^−4^ | 3.38×10^−1^ | 9.51×10^−2^ | 4.25×10^−3^ | 4.01×10^−2^ | 3.37×10^−1^ | 2.46×10^−5^ | 2.01×10^0^ |
|  | Zn | 3.38×10^−3^ | 1.70×10^−4^ | 1.72×10^−3^ | 1.19×10^−2^ | 1.20×10^−8^ | 7.38×10^−2^ | 3.98×10^−3^ | 3.96×10^−4^ | 2.60×10^−3^ | 1.20×10^−2^ | 3.15×10^−6^ | 1.85×10^−1^ |
|  | **Σ** | **1.02×10^−1^** |  |  |  |  |  | **1.20×10^−1^** |  |  |  |  |  |

**Supplementary Table S16 continued.** Statistical results for Hazard Quotient (HQ) and Hazard Index (HI) modelled values, representing non-carcinogenic health risks (NCHR) by exposure to trace metals in indoor dust for children. These results were derived from a probabilistic risk assessment encompassing 10,000 iterations for home indoor dust in the Illawarra region and indoor dust across Australian homes.

|  | **NCHR modelled in home indoor dust for children** | | | | | | | | | | | | |
| --- | --- | --- | --- | --- | --- | --- | --- | --- | --- | --- | --- | --- | --- |
|  |  | **Illawarra region homes** | | | | | | **Australian homes** | | | | | |
|  |  | **Mean** | **5%** | **Median** | **95%** | **Minimum** | **Maximum** | **Mean** | **5%** | **Median** | **95%** | **Minimum** | **Maximum** |
| **HQ Dermal** | As | 2.43×10^−3^ | 2.29×10^−4^ | 2.26×10^−3^ | 5.32×10^−3^ | 1.37×10^−7^ | 8.50×10^−3^ | 2.56×10^−3^ | 3.60×10^−5^ | 1.01×10^−3^ | 1.01×10^−2^ | 7.37×10^−8^ | 4.01×10^−1^ |
|  | Cu | 1.95×10^−2^ | 1.77×10^−3^ | 1.74×10^−2^ | 4.51×10^−2^ | 1.01×10^−6^ | 8.20×10^−2^ | 4.19×10^−3^ | 8.54×10^−5^ | 2.14×10^−3^ | 1.52×10^−2^ | 1.56×10^−7^ | 7.78×10^−2^ |
|  | Mn | 9.18×10^−3^ | 3.22×10^−4^ | 6.70×10^−3^ | 2.64×10^−2^ | 4.27×10^−7^ | 4.70×10^−2^ | 2.01×10^−4^ | 1.75×10^−6^ | 7.92×10^−5^ | 8.15×10^−4^ | 3.90×10^−10^ | 4.17×10^−3^ |
|  | Ni | 1.01×10^−3^ | 9.54×10^−5^ | 9.42×10^−4^ | 2.22×10^−3^ | 5.72×10^−8^ | 3.54×10^−3^ | 2.26×10^−3^ | 2.33×10^−5^ | 8.72×10^−4^ | 9.09×10^−3^ | 3.85×10^−8^ | 4.88×10^−2^ |
|  | Pb | 2.46×10^−2^ | 6.66×10^−4^ | 1.09×10^−2^ | 9.18×10^−2^ | 2.90×10^−6^ | 9.91×10^-2^ | 6.55×10^−2^ | 6.61×10^−4^ | 2.12×10^−2^ | 2.64×10^−1^ | 3.85×10^−7^ | 8.12×10^-1^ |
|  | Zn | 3.08×10^−3^ | 2.89×10^−4^ | 2.86×10^−3^ | 6.73×10^−3^ | 1.73×10^−7^ | 1.07×10^−2^ | 7.84×10^−3^ | 1.25×10^−4^ | 4.24×10^−3^ | 2.77×10^−2^ | 4.16×10^−8^ | 7.44×10^−2^ |
|  | **Σ** | **4.17×10^−2^** |  |  |  |  |  | **8.25×10^−2^** |  |  |  |  |  |
| **HQ Ingestion** | As | 6.69×10^−2^ | 6.05×10^−3^ | 5.97×10^−2^ | 1.55×10^−1^ | 3.47×10^−6^ | 2.81×10^−1^ | 7.31×10^−2^ | 1.04×10^−3^ | 2.88×10^−2^ | 2.86×10^−1^ | 2.14×10^−6^ | 1.05×10^0^ |
|  | Cu | 3.80×10^−2^ | 1.34×10^−3^ | 2.78×10^−2^ | 1.10×10^−1^ | 1.77×10^−6^ | 1.95×10^−1^ | 1.80×10^−2^ | 3.70×10^−4^ | 9.17×10^−3^ | 6.49×10^−2^ | 6.89×10^−7^ | 6.14×10^−2^ |
|  | Mn | 6.24×10^−2^ | 5.65×10^−3^ | 5.57×10^−2^ | 1.44×10^−1^ | 3.24×10^−6^ | 2.62×10^−1^ | 1.55×10^−3^ | 1.34×10^−5^ | 6.14×10^−4^ | 6.36×10^−3^ | 3.06×10^−9^ | 2.27×10^−1^ |
|  | Ni | 6.38×10^−2^ | 2.24×10^−3^ | 4.66×10^−2^ | 1.84×10^−1^ | 2.99×10^−6^ | 3.26×10^−1^ | 6.36×10^−2^ | 6.45×10^−4^ | 2.47×10^−2^ | 2.57×10^−1^ | 1.10×10^−6^ | 5.29×10^-2^ |
|  | Pb | 3.20×10^−1^ | 8.49×10^−3^ | 1.40×10^−1^ | 1.21×10^0^ | 4.95×10^−5^ | 7.11×10^-1^ | 4.71×10^−1^ | 4.67×10^−3^ | 1.51×10^−1^ | 1.90×10^0^ | 2.87×10^−6^ | 4.20×10^0^ |
|  | Zn | 2.65×10^−2^ | 2.40×10^−3^ | 2.36×10^−2^ | 6.12×10^−2^ | 1.37×10^−6^ | 1.11×10^−1^ | 3.61×10^−2^ | 5.61×10^−4^ | 1.95×10^−2^ | 1.28×10^−1^ | 1.99×10^−7^ | 3.43×10^−1^ |
|  | **Σ** | **4.96×10^−1^** |  |  |  |  |  | **6.63×10^−1^** |  |  |  |  |  |
| **HQ Inhalation** | As | 6.58×10^−4^ | 2.31×10^−5^ | 4.81×10^−4^ | 1.90×10^−3^ | 3.07×10^−8^ | 3.37×10^−3^ | 6.63×10^−4^ | 1.04×10^−5^ | 2.71×10^−4^ | 2.56×10^−3^ | 7.20×10^−9^ | 3.19×10^−1^ |
|  | Cu | 1.39×10^−3^ | 1.31×10^−4^ | 1.29×10^−3^ | 3.05×10^−3^ | 7.85×10^−8^ | 4.86×10^−3^ | 1.47×10^−3^ | 3.21×10^−5^ | 7.79×10^−4^ | 5.30×10^−3^ | 1.10×10^−8^ | 5.21×10^−2^ |
|  | Mn | 6.74×10^−6^ | 6.34×10^−7^ | 6.26×10^−6^ | 1.48×10^−5^ | 3.80×10^−10^ | 2.35×10^−5^ | 4.15×10^−6^ | 4.25×10^−8^ | 1.65×10^−6^ | 1.66×10^−5^ | 4.37×10^−11^ | 9.48×10^−4^ |
|  | Ni | 4.37×10^−4^ | 3.95×10^−5^ | 3.90×10^−4^ | 1.01×10^−3^ | 2.27E×10^−8^ | 1.84×10^−3^ | 5.42×10^−4^ | 6.33×10^−6^ | 2.20×10^−4^ | 2.20×10^−3^ | 5.23×10^−9^ | 2.38×10^−2^ |
|  | Pb | 3.74×10^−2^ | 5.13×10^−4^ | 1.37×10^−3^ | 1.50×10^−2^ | 3.45×10^−7^ | 2.44×10^-2^ | 6.88×10^−2^ | 7.52×10^−4^ | 2.26×10^−2^ | 2.74×10^−1^ | 5.16×10^−7^ | 7.89×10^-1^ |
|  | Zn | 7.73×10^−4^ | 2.72×10^−5^ | 5.64×10^−4^ | 2.22×10^−3^ | 3.60×10^−8^ | 3.96×10^−3^ | 1.36×10^−3^ | 2.24×10^−5^ | 7.52×10^−4^ | 4.85×10^−3^ | 2.35×10^−8^ | 1.20×10^−2^ |
|  | **Σ** | **4.07×10^−2^** |  |  |  |  |  | **7.28×10^−2^** |  |  |  |  |  |
| **HI** | As | 7.00×10^−2^ | 7.05×10^−3^ | 6.27×10^−2^ | 1.60×10^−1^ | 1.26×10^−4^ | 2.55×10^−1^ | 7.64×10^−2^ | 1.36×10^−3^ | 3.02×10^−2^ | 2.97×10^−1^ | 3.85×10^−5^ | 1.77×10^0^ |
|  | Cu | 5.90×10^−2^ | 1.19×10^−2^ | 5.08×10^−2^ | 1.33×10^−1^ | 8.01×10^−5^ | 2.07×10^−1^ | 2.37×10^−2^ | 1.09×10^−3^ | 1.27×10^−2^ | 8.29×10^−2^ | 1.30×10^−5^ | 2.16×10^−1^ |
|  | Mn | 7.16×10^−2^ | 1.26×10^−2^ | 6.51×10^−2^ | 1.55×10^−1^ | 2.19×10^−4^ | 2.66×10^−1^ | 1.75×10^−3^ | 1.67×10^−5^ | 6.99×10^−4^ | 7.17×10^−3^ | 5.16×10^−8^ | 3.89×10^−1^ |
|  | Ni | 6.53×10^−2^ | 3.66×10^−3^ | 4.80×10^−2^ | 8.85×10^−2^ | 1.57×10^−5^ | 9.26×10^−2^ | 6.64×10^−2^ | 8.28×10^−4^ | 2.59×10^−2^ | 2.67×10^−1^ | 7.89×10^−6^ | 2.12×10^-1^ |
|  | Pb | 3.48×10^−1^ | 1.04×10^−2^ | 1.54×10^−1^ | 4.32×10^-1^ | 5.79×10^−5^ | 7.40×10^-1^ | 6.05×10^−1^ | 1.52×10^−2^ | 2.17×10^−1^ | 2.38×10^0^ | 2.62×10^−4^ | 5.92×10^0^ |
|  | Zn | 3.03×10^−2^ | 3.54×10^−3^ | 2.73×10^−2^ | 6.85×10^−2^ | 9.21×10^−5^ | 8.22×10^−2^ | 4.53×10^−2^ | 1.30×10^−3^ | 2.51×10^−2^ | 1.58×10^−1^ | 4.21×10^−6^ | 4.17×10^−1^ |
|  | **Σ** | **6.44×10^−1^** |  |  |  |  |  | **8.18×10^−1^** |  |  |  |  |  |

**Supplementary Table S16 continued.** Statistical results for Hazard Quotient (HQ) and Hazard Index (HI) modelled values, representing non-carcinogenic health risks (NCHR) by exposure to trace metals in indoor dust for adults. These results were derived from a probabilistic risk assessment encompassing 10,000 iterations for home indoor dust in the Illawarra region and indoor dust across Australian homes.

|  | **NCHR modelled in home indoor dust for adults** | | | | | | | | | | | | |
| --- | --- | --- | --- | --- | --- | --- | --- | --- | --- | --- | --- | --- | --- |
|  |  | **Illawarra region homes** | | | | | | **Australian homes** | | | | | |
|  |  | **Mean** | **5%** | **Median** | **95%** | **Minimum** | **Maximum** | **Mean** | **5%** | **Median** | **95%** | **Minimum** | **Maximum** |
| **HQ Dermal** | As | 1.68×10^−3^ | 5.24×10^−5^ | 1.24×10^−3^ | 4.76×10^−3^ | 8.13×10^−9^ | 9.26×10^−3^ | 1.80×10^−3^ | 2.54×10^−5^ | 6.84×10^−4^ | 7.16×10^−3^ | 4.90×10^−8^ | 4.96×10^−2^ |
|  | Cu | 1.54×10^−2^ | 2.61×10^−4^ | 6.01×10^−3^ | 5.78×10^−2^ | 5.98×10^−8^ | 1.02×10^-1^ | 2.93×10^−3^ | 6.48×10^−5^ | 1.53×10^−3^ | 1.01×10^−2^ | 4.66×10^−8^ | 8.77×10^−2^ |
|  | Mn | 7.60×10^−4^ | 5.50×10^−5^ | 5.69×10^−4^ | 2.11×10^−3^ | 1.02×10^−7^ | 1.15×10^−2^ | 3.15×10^−4^ | 2.65×10^−6^ | 1.22×10^−4^ | 1.27×10^−3^ | 1.23×10^−9^ | 7.87×10^−3^ |
|  | Ni | 2.51×10^−3^ | 7.83×10^−5^ | 1.86×10^−3^ | 7.13×10^−3^ | 1.22×10^−8^ | 1.38×10^−2^ | 1.97×10^−3^ | 1.99×10^−5^ | 7.60×10^−4^ | 7.80×10^−3^ | 1.11×10^−8^ | 4.90×10^−2^ |
|  | Pb | 3.21×10^−2^ | 7.45×10^−4^ | 1.71×10^−2^ | 1.86×10^−1^ | 3.77×10^−6^ | 5.01×10^-1^ | 5.95×10^−2^ | 6.76×10^−4^ | 1.92×10^−2^ | 2.34×10^−1^ | 6.82×10^−7^ | 8.72×10^-1^ |
|  | Zn | 7.62×10^−3^ | 2.38×10^−4^ | 5.64×10^−3^ | 2.16×10^−2^ | 3.69×10^−8^ | 4.20×10^−2^ | 7.23×10^−3^ | 9.66×10^−5^ | 3.82×10^−3^ | 2.58×10^−2^ | 1.32×10^−7^ | 6.08×10^−2^ |
|  | **Σ** | **6.01×10^−2^** |  |  |  |  |  | **7.38×10^−2^** |  |  |  |  |  |
| **HQ Ingestion** | As | 5.96×10^−2^ | 1.81×10^−3^ | 4.39×10^−2^ | 1.69×10^−1^ | 2.74×10^−7^ | 3.26×10^−1^ | 6.13×10^−2^ | 8.42×10^−4^ | 2.30×10^−2^ | 2.44×10^−1^ | 2.03×10^−6^ | 1.01×10^0^ |
|  | Cu | 2.20×10^−2^ | 9.24×10^−4^ | 1.02×10^−2^ | 7.94×10^−2^ | 3.73×10^−6^ | 1.20×10^-1^ | 1.27×10^−2^ | 2.77×10^−4^ | 6.54×10^−3^ | 4.48×10^−2^ | 2.36×10^−7^ | 3.55×10^−1^ |
|  | Mn | 2.61×10^−2^ | 1.76×10^−3^ | 1.91×10^−2^ | 7.40×10^−2^ | 3.37×10^−6^ | 4.47×10^−1^ | 1.08×10^−3^ | 9.16×10^−6^ | 4.13×10^−4^ | 4.39×10^−3^ | 4.94×10^−9^ | 2.69×10^−2^ |
|  | Ni | 6.30×10^−3^ | 1.92×10^−4^ | 4.64×10^−3^ | 1.78×10^−2^ | 2.90×10^−8^ | 3.44×10^−2^ | 1.56×10^−2^ | 1.59×10^−4^ | 5.85×10^−3^ | 6.28×10^−2^ | 5.89×10^−8^ | 4.17×10^−1^ |
|  | Pb | 1.12×10^−1^ | 1.26×10^−3^ | 3.92×10^−2^ | 4.38×10^−1^ | 9.39×10^−7^ | 6.10×10^-1^ | 3.91×10^−1^ | 4.28×10^−3^ | 1.22×10^−1^ | 1.57×10^0^ | 5.18×10^−6^ | 2.56×10^0^ |
|  | Zn | 1.83×10^−2^ | 6.28×10^−4^ | 1.35×10^−2^ | 5.22×10^−2^ | 7.12×10^−8^ | 8.28×10^−2^ | 2.49×10^−2^ | 3.30×10^−4^ | 1.28×10^−2^ | 9.03×10^−2^ | 3.74×10^−7^ | 2.53×10^−1^ |
|  | **Σ** | **2.27×10^−1^** |  |  |  |  |  | **5.06×10^−1^** |  |  |  |  |  |
| **HQ Inhalation** | As | 5.08×10^−4^ | 1.74×10^−5^ | 3.73×10^−4^ | 1.45×10^−3^ | 1.97×10^−9^ | 2.29×10^−3^ | 5.92×10^−4^ | 8.24×10^−6^ | 2.35×10^−4^ | 2.29×10^−3^ | 3.19×10^−9^ | 2.49×10^−2^ |
|  | Cu | 2.89×10^−3^ | 1.23×10^−4^ | 1.37×10^−3^ | 1.04×10^−2^ | 5.12×10^−7^ | 1.36×10^−1^ | 1.64×10^−3^ | 3.57×10^−5^ | 8.48×10^−4^ | 5.86×10^−3^ | 3.95×10^−8^ | 5.10×10^−2^ |
|  | Mn | 6.35×10^−6^ | 1.77×10^−7^ | 3.89×10^−6^ | 2.04×10^−5^ | 9.90×10^−11^ | 9.69×10^−5^ | 3.25×10^−6^ | 3.23×10^−8^ | 1.32×10^−6^ | 1.31×10^−5^ | 3.99×10^−12^ | 7.81×10^−5^ |
|  | Ni | 3.57×10^−4^ | 1.23×10^−5^ | 2.63×10^−4^ | 1.02×10^−3^ | 1.39×10^−9^ | 1.61×10^−3^ | 5.14×10^−4^ | 5.31×10^−6^ | 1.99×10^−4^ | 2.05×10^−3^ | 7.57×10^−11^ | 2.02×10^−2^ |
|  | Pb | 3.72×10^−2^ | 9.56×10^−4^ | 2.24×10^−2^ | 2.46×10^−1^ | 4.98×10^−6^ | 4.23×10^-1^ | 6.89×10^−2^ | 7.89×10^−4^ | 2.30×10^−2^ | 2.75×10^−1^ | 1.99×10^−8^ | 1.48×10^0^ |
|  | Zn | 9.56×10^−4^ | 2.91×10^−5^ | 7.03×10^−4^ | 2.70×10^−3^ | 4.39×10^−9^ | 5.22×10^−3^ | 1.21×10^−3^ | 1.80×10^−5^ | 6.49×10^−4^ | 4.31×10^−3^ | 3.76×10^−9^ | 9.84×10^−3^ |
|  | **Σ** | **4.19×10^−2^** |  |  |  |  |  | **7.28×10^−2^** |  |  |  |  |  |
| **HI** | As | 6.18×10^−2^ | 2.45×10^−3^ | 4.55×10^−2^ | 1.74×10^−1^ | 4.44×10^−5^ | 2.35×10^−1^ | 6.37×10^−2^ | 1.13×10^−3^ | 2.40×10^−2^ | 2.52×10^−1^ | 8.24×10^−6^ | 1.16×10^0^ |
|  | Cu | 3.82×10^−2^ | 3.23×10^−3^ | 1.85×10^−2^ | 1.15×10^−1^ | 7.35×10^−5^ | 1.94×10^-1^ | 1.73×10^−2^ | 9.52×10^−4^ | 9.55×10^−3^ | 5.76×10^−2^ | 3.77×10^−6^ | 4.94×10^−1^ |
|  | Mn | 2.69×10^−2^ | 1.83×10^−3^ | 1.97×10^−2^ | 7.61×10^−2^ | 7.05×10^−6^ | 2.07×10^−1^ | 1.40×10^−3^ | 1.28×10^−5^ | 5.42×10^−4^ | 5.68×10^−3^ | 9.52×10^−8^ | 3.31×10^−2^ |
|  | Ni | 9.17×10^−3^ | 6.18×10^−4^ | 6.84×10^−3^ | 2.54×10^−2^ | 1.29×10^−5^ | 3.78×10^−2^ | 1.81×10^−2^ | 3.00×10^−4^ | 7.08×10^−3^ | 7.18×10^−2^ | 6.52×10^−6^ | 4.64×10^−1^ |
|  | Pb | 1.81×10^−1^ | 9.02×10^−3^ | 1.02×10^−1^ | 4.06×10^−1^ | 1.11×10^−4^ | 6.22×10^-1^ | 5.19×10^−1^ | 1.44×10^−2^ | 1.85×10^−1^ | 2.00×10^0^ | 2.92×10^−4^ | 4.36×10^0^ |
|  | Zn | 2.69×10^−2^ | 3.76×10^−3^ | 2.34×10^−2^ | 6.24×10^−2^ | 1.32×10^−4^ | 7.18×10^−2^ | 3.33×10^−2^ | 7.59×10^−4^ | 1.80×10^−2^ | 1.19×10^−1^ | 2.45×10^−6^ | 3.10×10^−1^ |
|  | **Σ** | **3.29×10^−1^** |  |  |  |  |  | **6.53×10^−1^** |  |  |  |  |  |

**Supplementary Table S17.** Statistical results for carcinogenic risk (CR) modelled values, by exposure to trace metals in garden soils for children and adults. These results were derived from a probabilistic risk assessment encompassing 10,000 iterations for home indoor dust in the Illawarra region and indoor dust across Australian homes. CR was calculated for As and Pb due to their carcinogenicity potential and the availability of SF.

|  | **CR modelled in home garden soil for children** | | | | | | | | | | | | |
| --- | --- | --- | --- | --- | --- | --- | --- | --- | --- | --- | --- | --- | --- |
|  |  | **Illawarra region** | | | | | | **Australia** | | | | | |
|  |  | Mean | 5% | Median | 95% | Minimum | Maximum | Mean | 5% | Median | 95% | Minimum | Maximum |
| **Dermal** | As | 2.27×10^−7^ | 7.41×10^−9^ | 1.26×10^−7^ | 7.72×10^−7^ | 1.12×10^−10^ | 4.72×10^−6^ | 2.29×10^−7^ | 1.13×10^−8^ | 8.28×10^−8^ | 9.84×10^−7^ | 4.14×10^−9^ | 2.56×10^−5^ |
|  | **Σ** | **2.27×10^−7^** |  |  |  |  |  | **2.29×10^−7^** |  |  |  |  |  |
| **Ingestion** | As | 2.16×10^−5^ | 6.78×10^−7^ | 1.18×10^−5^ | 7.46×10^−5^ | 1.81×10^−10^ | 5.53×10^−5^ | 2.27×10^−5^ | 2.66×10^−7^ | 7.47×10^−6^ | 8.80×10^−5^ | 4.10×10^−8^ | 3.10×10^−5^ |
|  | Pb | 2.10×10^−6^ | 1.90×10^−7^ | 1.88×10^−6^ | 4.86×10^−6^ | 1.09×10^−10^ | 1.08×10^−5^ | 2.33×10^−6^ | 1.03×10^−8^ | 4.97×10^−7^ | 9.76×10^−6^ | 2.78×10^−10^ | 4.02×10^−5^ |
|  | **Σ** | **2.37×10^−5^** |  |  |  |  |  | **2.30×10^−5^** |  |  |  |  |  |
| **Inhalation** | As | 1.80×10^−6^ | 2.44×10^−8^ | 8.24×10^−7^ | 6.92×10^−6^ | 2.83×10^−10^ | 6.05×10^−5^ | 1.83×10^−6^ | 5.44×10^−10^ | 6.41×10^−7^ | 7.87×10^−6^ | 3.19×10^−10^ | 2.66×10^−4^ |
|  | Pb | 2.10×10^−6^ | 7.38×10^−8^ | 1.53×10^−6^ | 6.04×10^−6^ | 9.78×10^−11^ | 1.08×10^−5^ | 2.43×10^−6^ | 9.09×10^−9^ | 3.38×10^−7^ | 8.96×10^−6^ | 7.58×10^−10^ | 7.29×10^−5^ |
|  | **Σ** | **3.90×10^−6^** |  |  |  |  |  | **4.27×10^−6^** |  |  |  |  |  |
|  | **Total Risk** | **2.78×10^−5^** |  |  |  |  |  | **2.95×10^−5^** |  |  |  |  |  |
|  | **CR modelled in home garden soil for adult** | | | | | | | | | | | | |
|  |  | **Illawarra region** | | | | | | **Australia** | | | | | |
|  |  | Mean | 5% | Median | 95% | Minimum | Maximum | Mean | 5% | Median | 95% | Minimum | Maximum |
| **Dermal** | As | 7.71×10^−8^ | 1.38×10^−9^ | 3.39×10^−8^ | 2.88×10^−7^ | 8.83×10^−11^ | 2.68×10^−6^ | 8.08×10^−8^ | 1.23×10^−9^ | 3.90×10^−8^ | 3.15×10^−7^ | 4.14×10^−11^ | 1.23×10^−5^ |
|  | **Σ** | **7.71×10^−8^** |  |  |  |  |  | **8.08×10^−8^** |  |  |  |  |  |
| **Ingestion** | As | 4.68×10^−6^ | 5.36×10^−8^ | 2.06×10^−6^ | 1.77×10^−5^ | 3.07×10^−8^ | 1.02×10^−4^ | 5.28×10^−6^ | 1.24×10^−7^ | 2.48×10^−6^ | 2.08×10^−5^ | 5.92×10^−9^ | 9.54×10^−5^ |
|  | Pb | 1.31×10^−6^ | 4.08×10^−8^ | 9.69×10^−7^ | 3.71×10^−6^ | 4.34×10^−12^ | 7.22×10^−6^ | 1.57×10^−6^ | 1.33×10^−8^ | 3.92×10^−7^ | 6.43×10^−6^ | 3.92×10^−10^ | 3.12×10^−5^ |
|  | **Σ** | **5.99×10^−6^** |  |  |  |  |  | **6.86×10^−6^** |  |  |  |  |  |
| **Inhalation** | As | 2.03×10^−6^ | 3.74×10^−8^ | 8.98×10^−7^ | 7.57×10^−6^ | 1.08×10^−10^ | 7.56×10^−5^ | 2.31×10^−6^ | 4.26×10^−8^ | 7.93×10^−7^ | 1.00×10^−5^ | 4.40×10^−11^ | 5.54×10^−5^ |
|  | Pb | 1.51×10^−6^ | 4.59×10^−8^ | 1.11×10^−6^ | 4.27×10^−6^ | 8.94×10^−14^ | 8.24×10^−6^ | 1.48×10^−6^ | 5.53×10^−10^ | 2.72×10^−7^ | 5.83×10^−6^ | 1.63×10^−11^ | 5.23×10^−5^ |
|  | **Σ** | **3.54×10^−6^** |  |  |  |  |  | **3.79×10^−6^** |  |  |  |  |  |
|  | **Total Risk** | **9.61×10^−6^** |  |  |  |  |  | **1.07×10^−5^** |  |  |  |  |  |

**Supplementary Table S17 continued.** Statistical results for carcinogenic risk (CR) modelled values by exposure to trace metals in indoor dust for children and adults. These results were derived from a probabilistic risk assessment encompassing 10,000 iterations for home indoor dust in the Illawarra region and indoor dust across Australian homes. CR was calculated for As and Pb due to their carcinogenicity potential and the availability of SF.

|  |  | **CR modelled in home indoor dust for children** | | | | | | | | | | | |
| --- | --- | --- | --- | --- | --- | --- | --- | --- | --- | --- | --- | --- | --- |
|  |  | **Illawarra region** | | | | | | **Australia** | | | | | |
|  |  | Mean | 5% | Median | 95% | Minimum | Maximum | Mean | 5% | Median | 95% | Minimum | Maximum |
| **Dermal** | As | 1.10×10^−6^ | 1.03×10^−7^ | 1.02×10^−6^ | 2.40×10^−6^ | 6.18×10^−11^ | 3.83×10^−6^ | 1.15×10^−6^ | 1.62×10^−8^ | 4.53×10^−7^ | 4.53×10^−6^ | 3.32×10^−11^ | 1.81×10^−4^ |
|  | **Σ** | **1.10×10^−6^** |  |  |  |  |  | **1.15×10^−6^** |  |  |  |  |  |
| **Ingestion** | As | 3.01×10^−5^ | 5.44×10^−10^ | 5.38×10^−9^ | 1.39×10^−8^ | 3.13×10^−13^ | 1.43×10^−5^ | 3.29×10^−5^ | 4.70×10^−7^ | 1.29×10^−5^ | 1.29×10^−4^ | 9.63×10^−10^ | 4.73×10^−4^ |
|  | Pb | 9.52×10^−6^ | 2.53×10^−7^ | 4.18×10^−6^ | 3.61×10^−5^ | 1.47×10^−9^ | 4.78×10^−4^ | 1.40×10^−5^ | 1.39×10^−7^ | 4.50×10^−6^ | 5.65×10^−5^ | 8.54×10^−11^ | 1.25×10^−4^ |
|  | **Σ** | **3.96×10^−5^** |  |  |  |  |  | **4.69×10^−5^** |  |  |  |  |  |
| **Inhalation** | As | 2.98×10^−6^ | 1.05×10^−7^ | 2.18×10^−6^ | 8.59×10^−6^ | 1.39×10^−10^ | 1.53×10^−5^ | 3.00×10^−6^ | 4.72×10^−8^ | 1.23×10^−6^ | 1.16×10^−5^ | 3.26×10^−11^ | 1.45×10^−3^ |
|  | Pb | 5.53×10^−6^ | 7.58×10^−8^ | 2.03×10^−6^ | 2.22×10^−5^ | 5.11×10^−11^ | 2.13×10^−4^ | 9.88×10^-6^ | 1.11×10^−7^ | 3.34×10^−6^ | 4.05×10^−5^ | 7.63×10^−11^ | 1.17×10^−4^ |
|  | **Σ** | **8.51×10^−6^** |  |  |  |  |  | **1.32×10^−5^** |  |  |  |  |  |
|  | **Total Risk** | **4.43×10^−5^** |  |  |  |  |  | **6.09×10^−5^** |  |  |  |  |  |
|  |  | **CR modelled in home indoor dust for adult** | | | | | | | | | | | |
|  |  | **Illawarra region** | | | | | | **Australia** | | | | | |
|  |  | Mean | 5% | Median | 95% | Minimum | Maximum | Mean | 5% | Median | 95% | Minimum | Maximum |
| **Dermal** | As | 7.56×10^−7^ | 2.36×10^−8^ | 5.60×10^−7^ | 2.14×10^−6^ | 3.66×10^−12^ | 4.17×10^−6^ | 8.10×10^−7^ | 1.14×10^−8^ | 3.08×10^−7^ | 3.22×10^−6^ | 1.83×10^−11^ | 2.23×10^−5^ |
|  | **Σ** | **7.56×10^−7^** |  |  |  |  |  | **8.10×10^−7^** |  |  |  |  |  |
| **Ingestion** | As | 2.68×10^−5^ | 8.16×10^−7^ | 1.97×10^−5^ | 7.59×10^−5^ | 1.23×10^−10^ | 1.47×10^−4^ | 2.76×10^−5^ | 3.23×10^−7^ | 1.04×10^−5^ | 1.10×10^−4^ | 7.80×10^−10^ | 4.55×10^−4^ |
|  | Pb | 3.32×10^−6^ | 3.76×10^−8^ | 1.17×10^−6^ | 1.30×10^−5^ | 2.79×10^−11^ | 1.82×10^−5^ | 1.16×10^−5^ | 1.27×10^−7^ | 3.63×10^−6^ | 4.67×10^−5^ | 3.75×10^−10^ | 7.62×10^−5^ |
|  | **Σ** | **3.01×10^−5^** |  |  |  |  |  | **3.92×10^−5^** |  |  |  |  |  |
| **Inhalation** | As | 2.30×10^−6^ | 7.86×10^−8^ | 1.69×10^−6^ | 6.55×10^−6^ | 8.94×10^−12^ | 1.04×10^−5^ | 2.68×10^−6^ | 3.73×10^−8^ | 1.67×10^−6^ | 1.63×10^−5^ | 2.27×10^−10^ | 1.77×10^−4^ |
|  | Pb | 9.18×10^−7^ | 1.41×10^−7^ | 3.31×10^−6^ | 3.64×10^−5^ | 7.37×10^−10^ | 6.22×0^−4^ | 1.02×10^−6^ | 1.17×10^−7^ | 4.06×10^−6^ | 4.84×10^−5^ | 3.51×10^−11^ | 2.19×10^−4^ |
|  | **Σ** | **3.22×10^−6^** |  |  |  |  |  | **3.70×10^−6^** |  |  |  |  |  |
|  | **Total Risk** | **3.41×10^−5^** |  |  |  |  |  | **4.37×10^−5^** |  |  |  |  |  |

**References**

Aendo, P., Netvichian, R., Thiendedsakul, P., Khaodhiar, S., & Tulayakul, P. (2022). Carcinogenic Risk of Pb, Cd, Ni, and Cr and Critical Ecological Risk of Cd and Cu in Soil and Groundwater around the Municipal Solid Waste Open Dump in Central Thailand. *Journal of environmental and public health*, *2022*.

Dancey, C. P., & Reidy, J. (2007). *Statistics without maths for psychology*. Pearson education.

DIGS. (2022a). *1:50 000 Kiama Geological map*. Digital Imaging Geological System, NSW government. Retrieved 25th January 2023 from https://search.geoscience.nsw.gov.au/report/R00030442

DIGS. (2022b). *1:100 000 Wollongong-Port Hacking Geological map*. Digital Imaging Geological System, NSW government. Retrieved 25th January 2023 from https://search.geoscience.nsw.gov.au/report/R00027834

Doyi, I. N. Y., Isley, C. F., Soltani, N. S., & Taylor, M. P. (2019). Human exposure and risk associated with trace element concentrations in indoor dust from Australian homes. *Environment International*, *133*, 105125. https://doi.org/https://doi.org/10.1016/j.envint.2019.105125

enHealth. (2012). *Australian Exposure Factor Guidance. Guidelines for assessing human health risks from environmental hazards*. Environmental Health Risk Management Retrieved 8 June 2023 from https://www.eh.org.au/documents/item/915

Fay, M. (2005). *Toxicological profile for nickel*. Agency for Toxic Substances and Disease Registry.

Fry, K., Gillings, M., Isley, C., Gunkel-Grillon, P., & Taylor, M. P. (2021). Trace element contamination of soil and dust by a New Caledonian ferronickel smelter: Dispersal, enrichment, and human health risk. *Environmental Pollution*, *288*, 117593.

Goumenou, M., & Tsatsakis, A. (2019). Proposing new approaches for the risk characterisation of single chemicals and chemical mixtures: The source related Hazard Quotient (HQ(S)) and Hazard Index (HI(S)) and the adversity specific Hazard Index (HI(A)). *Toxicol Rep*, *6*, 632-636. https://doi.org/10.1016/j.toxrep.2019.06.010

Hernández-Mena, L., Panduro-Rivera, M. G., Díaz-Torres, J. d. J., Ojeda-Castillo, V., Real-Olvera, J. d., López-Cervantes, M., Pacheco-Domínguez, R. L., Morton-Bermea, O., Santacruz-Benítez, R., & Vallejo-Rodríguez, R. (2021). GIS, multivariate statistics analysis and health risk assessment of water supply quality for human use in Central Mexico. *Water*, *13*(16), 2196.

Ibañez-Del Rivero, C., Fry, K. L., Gillings, M. M., Barlow, C. F., Aelion, C. M., & Taylor, M. P. (2023). Sources, pathways and concentrations of potentially toxic trace metals in home environments. *Environmental Research*, *220*, 115173. https://doi.org/https://doi.org/10.1016/j.envres.2022.115173

IRIS. (1985). *Integrated Risk Information System*. United States Environmental Protection Agency. Retrieved 21/July/2022 from https://iris.epa.gov/AtoZ/?list_type=alpha

Isbell, R. (2016). *The Australian soil classification*. CSIRO publishing.

Ju, Q., Hu, Y., Liu, Q., Chai, H., Chen, K., Zhang, H., & Wu, Y. (2023). Source apportionment and ecological health risks assessment from major ions, metalloids and trace elements in multi-aquifer groundwater near the Sunan mine area, Eastern China. *Science of The Total Environment*, *860*, 160454.

Li, H., Qian, X., Hu, W., Wang, Y., & Gao, H. (2013). Chemical speciation and human health risk of trace metals in urban street dusts from a metropolitan city, Nanjing, SE China. *Science of the Total Environment*, *456*, 212-221.

NEPC. (2011). National Environment Protection Measure (Assessment of Site Contamination) Schedule B4 - Guideline on site-specific health risk assessments. In (pp. 87p): National Environment Council.

NEPC. (2013). *National Environment Protection (Assessment of Site Contamination) Measure 1999, amended 2013. Canberra: National Environment Protection Council, Australian Government*. National Protection Council. Retrieved 16th July 2022 from https://www.legislation.gov.au/Details/F2012C00858

Norris, G. R., Duvall, S., Brown, S. B., Ohmsen, G., Chenhall, B., & Jones, B. (2014). *Positive Matrix Factorization (PMF) 5.0 Fundamentals and User Guide.*

OEHHA, C. (1999). Appendix D. 3 Chronic RELs and toxicity summaries using the previous version of the Hot Spots Risk Assessment Guidelines. In: California Office of Environmental Health Hazard Assessment.

Panqing, Y., Abliz, A., Xiaoli, S., & Aisaiduli, H. (2023). Human health-risk assessment of heavy metal–contaminated soil based on Monte Carlo simulation. *Scientific reports*, *13*(1), 7033.

Petit, J. C., Maggi, P., Pirard, C., Charlier, C., Ruttens, A., Liénard, A., Colinet, G., & Remy, S. (2022). Human biomonitoring survey (Pb, Cd, As, Cu, Zn, Mo) for urban gardeners exposed to metal contaminated soils. *Environmental Pollution*, *312*, 120028.

SEED. (2022). *The Central Resource for Sharing and Enabling Environmental Data in NSW*. NSW Government. Retrieved 28th January 2022 from https://www.seed.nsw.gov.au/

Taloor, A. K., Bala, A., & Mehta, P. (2023). Human health risk assessment and pollution index of groundwater in Jammu plains of India: A geospatial approach. *Chemosphere*, *313*, 137329.

USEPA. (2007). *SW-846 Test Method 6200: Field Portable X-Ray Fluorescence Spectrometry for the Determination of Elemental Concentrations in Soil and Sediment*. United States Environmetal Protection Agency. Retrieved 15 February 2022 from https://www.epa.gov/sites/default/files/2015-12/documents/6200.pdf

USEPA. (2011). Exposure factors handbook: 2011 edition. *United States Environmental Protection Agency*.

Yang, S., Zhao, J., Chang, S. X., Collins, C., Xu, J., & Liu, X. (2019). Status assessment and probabilistic health risk modeling of metals accumulation in agriculture soils across China: A synthesis. *Environment International*, *128*, 165-174.
